# Supplementary material for: Single versus combination treatment in tinnitus: an international, multicentre, parallel-arm, superiority, randomised controlled trial
Source: Nat Commun. 2025 Nov 21;16:10510. doi: 10.1038/s41467-025-66165-1 (PMC12647723; doi:10.1038/s41467-025-66165-1)
Supplement: Supplementary file 1 — Supplementary Information [file 41467_2025_66165_MOESM1_ESM.pdf]

Single versus Combination Treatment in Tinnitus: An International, Multicentre, Parallel-arm, Superiority, Randomised Controlled Trial

Table Of Contents for Supplementary Appendix

1. FIGURE S1: EXTENDED PATIENTS FLOWCHART (INTENTION-TO-TREAT AND PER-PROTOCOL SAMPLE) 4

2. TABLE S1: EXCLUDED PATIENTS FROM STUDY PARTICIPATION 5

3. TABLE S2: QUANTITY AND REASONS OF DROPOUTS BEFORE TREATMENT START PER TREATMENT ARM 6

4. TABLE S3: QUANTITY AND REASONS OF DROPOUTS DURING TREATMENT PER TREATMENT ARM – BEFORE INTERIM VISIT 7

5. TABLE S4: QUANTITY AND REASONS OF DROPOUTS DURING TREATMENT PER TREATMENT ARM – AFTER INTERIM VISIT 8

6. TABLE S5: QUANTITY AND REASONS OF PATIENTS LOST TO FOLLOW-UP 9

7. TABLE S6: DEMOGRAPHIC AND CLINICAL CHARACTERISTICS OF THE PARTICIPANTS AT BASELINE (STRATIFIED BY TREATMENT ARM). 10

8. FIGURE S2: AUDIOGRAM IN PATIENTS WITH AND WITHOUT HEARING AID INDICATION FOR SINGLE AND COMBINED TREATMENT GROUPS 12

9. FIGURE S3: AUDIOGRAM FOR PATIENTS IN ALL TREATMENT GROUPS 13

10. TABLE S7: REPRESENTATIVENESS OF STUDY PARTICIPANTS 14

11. TABLE S8: PRIMARY OUTCOME (THI) OBJECTIVE 1: MODEL PARAMETERS 15

12. FIGURE S4: PRIMARY OUTCOME (THI) OBJECTIVE 1: MODEL ASSUMPTIONS 16

13. FIGURE S5: PRIMARY OUTCOME (THI) OBJECTIVE 2 17

14. TABLE S9: THI CHANGE FROM BASELINE TO FINAL VISIT FOR HEARING AID INDICATION (YES/NO) 18

15. TABLE S10: THI CHANGE FROM BASELINE TO FINAL VISIT FOR HIGH AND LOW TINNITUS DISTRESS SEVERITY 19

16. TABLE S11: PRIMARY OUTCOME (THI) OBJECTIVE 4 & 5 AT FINAL VISIT 20

17. TABLE S12: PRIMARY OUTCOME (THI) AT INTERIM VISIT 21

18. TABLE S13: PRIMARY OUTCOME (THI) AT FOLLOW-UP 22

19. TABLE S14: THI CHANGE FROM BASELINE TO FINAL VISIT FOR EACH COUNTRY 23

20. TABLE S15: SECONDARY OUTCOME (TFI) OBJECTIVE 4 & 5 AT FINAL VISIT 24

21. TABLE S16: SECONDARY OUTCOME (MINI-TQ) OBJECTIVE 4 & 5 AT FINAL VISIT 25

22. TABLE S17: SECONDARY OUTCOME (NRS-2: "HOW STRONG OR LOUD IS YOUR TINNITUS AT PRESENT?") OBJECTIVE 4 & 5 AT FINAL VISIT........ 26

23. TABLE S18: SECONDARY OUTCOME (PHQ-9) OBJECTIVE 4 & 5 AT FINAL VISIT........ 27

24. TABLE S19: SECONDARY OUTCOME (WHO-QOL-BREF 1: PHYSICAL HEALTH) AT FINAL... 28

VISIT........ 28

25. TABLE S20: SECONDARY OUTCOME (WHO-QOL-BREF 2: PSYCHOLOGICAL HEALTH) AT FINAL VISIT........ 29

26. TABLE S21: SECONDARY OUTCOME (WHO-QOL-BREF 3: SOCIAL FACTORS) AT FINAL...... 30

VISIT........ 30

27. TABLE S22: SECONDARY OUTCOME (WHO-QOL-BREF 4: ENVIRONMENT) AT FINAL........ 31

VISIT........ 31

28. TABLE S23: SECONDARY OUTCOME (NRS 1: "HOW MUCH OF A PROBLEM IS YOUR TINNITUS AT PRESENT?") AT FINAL VISIT........ 32

29. TABLE S24: SECONDARY OUTCOME (NRS 3: "HOW UNCOMFORTABLE IS YOUR TINNITUS AT PRESENT?") AT FINAL VISIT........ 33

30. TABLE S25: SECONDARY OUTCOME (NRS 4: "HOW ANNOYING IS YOUR TINNITUS AT PRESENT?") AT FINAL VISIT........ 34

31. TABLE S26: SECONDARY OUTCOME (NRS 5: "HOW EASY IS IT FOR YOU TO IGNORE YOUR TINNITUS AT PRESENT?") AT FINAL VISIT........ 35

32. TABLE S27: SECONDARY OUTCOME (NRS 6: "HOW UNPLEASANT IS YOUR TINNITUS AT PRESENT?") AT FINAL VISIT........ 36

33. FIGURE S6: SECONDARY OUTCOME (CGI-I) AT FINAL VISIT – SINGLE VS. COMBINATION (OBJECTIVE 1)........ 37

34. FIGURE S7: SECONDARY OUTCOME (CGI-I) AT FINAL VISIT – ALL TREATMENT ARMS (OBJECTIVE 2)........ 38

35. FIGURE S8: SECONDARY OUTCOME (CGI-I) AT FINAL VISIT – ALL TREATMENT ARMS (OBJECTIVE 2) IN PATIENTS WITH AND WITHOUT HEARING AID INDICATION........ 39

36. FIGURE S9: SECONDARY OUTCOME (CGI-I) AT FINAL VISIT – ALL TREATMENT ARMS (OBJECTIVE 2) IN PATIENTS WITH HIGH AND LOW TINNITUS DISTRESS SEVERITY........ 40

37. TABLE S28: QUANTITY AND TYPE OF ADVERSE EVENTS PER TREATMENT ARM........ 41

38. TABLE S29: COMPLIANCE TO TREATMENTS........ 43

39. TABLE S30: PRIMARY OUTCOME (THI) OBJECTIVE 2: POST-HOC COMPARISONS (BASELINE TO FINAL VISIT)........ 44

40. TABLE S31: SENSITIVITY ANALYSIS - PRIMARY OUTCOME (THI) AT FINAL VISIT........ 46

41. TABLE S32: SENSITIVITY ANALYSIS – ROBUSTNESS CHECK OF THE PRIMARY OUTCOME (THI) AT FINAL VISIT USING DIFFERENT IMPUTATION METHODS 47

42. FIGURE S10: PER PROTOCOL (N = 185) PRIMARY OUTCOME (THI) 48

43. TABLE S33: PER PROTOCOL (N = 185) PRIMARY OUTCOME (THI) AT FINAL VISIT 49

44. TABLE S34: PER PROTOCOL 2 (N = 155) PRIMARY OUTCOME (THI) AT FINAL VISIT 50

45. TABLE S35: CLINICAL SITES AND PRINCIPAL INVESTIGATORS 51

46. TABLE S36: AUTHOR CONTRIBUTIONS 52

47. TABLE S37: CRITERIA FOR HA INDICATION 54

48. TABLE S38: RESPONSIBLE PERSONS FOR THE CONCEPTION OF TREATMENTS 55

49. FIGURE S11: BOX-AND-WHISKER PLOT OF OBSERVED AND IMPUTED THI VALUES 56

50. REFERENCES 57

51. PUBLISHED STUDY PROTOCOL & STATISTICAL ANALYSIS PLAN 58

52. ETHICS APPROVALS 59

1. *Figure S1: Extended Patients Flowchart (Intention-to-treat and Per-protocol Sample)*

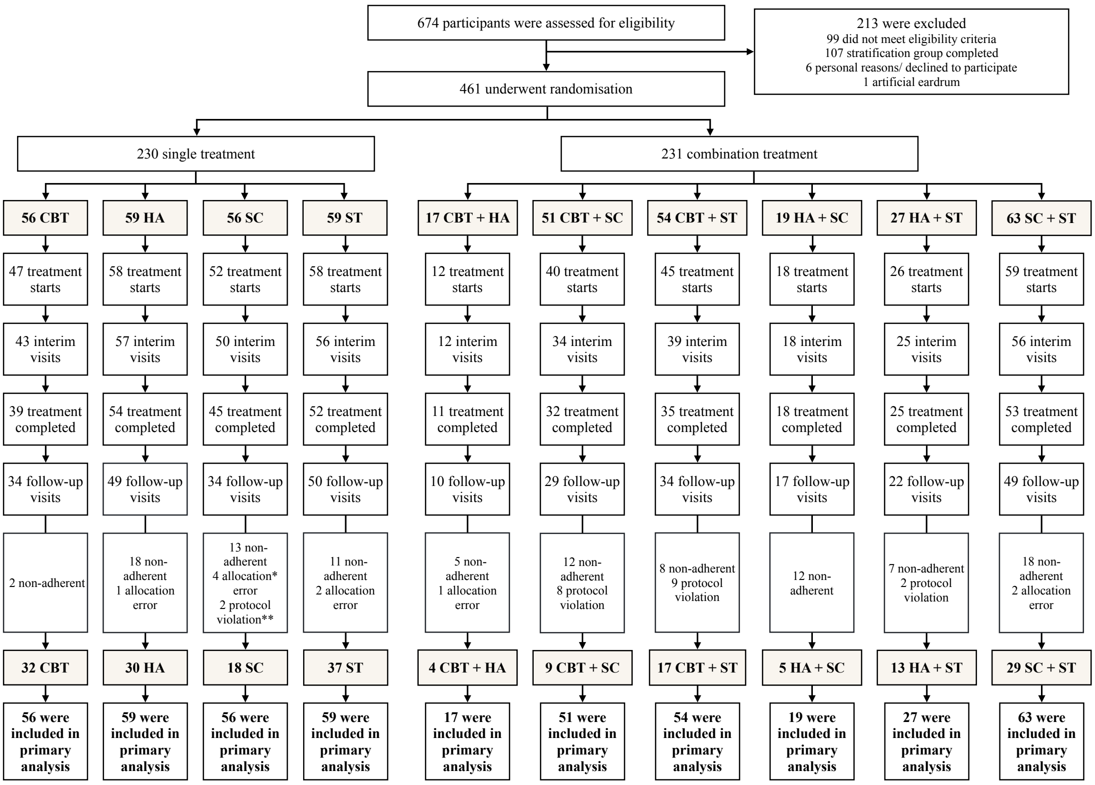

Note. A total of 674 patient were screened of whom 461 (ITT sample) met the trial inclusion criteria and were randomly assigned to one of ten treatment arms comprised of a single treatment or a combination of two treatments using a set of four different therapy approaches - cognitive-behavioural therapy (CBT), hearing aids (HA), structured counselling (SC), and sound therapy (ST). 230 were assigned to single treatments and 231 were assigned to combination treatments. 213 subjects were excluded during the screening process. Available data for the primary outcome (incl. missing data) per study visit and treatment arm can be seen. The number of treatment non-adherence, protocol violations and allocation errors are equally shown per treatment arm. From the sample of 194 patients for the per-protocol analysis, nine had missings in covariates relevant for the analysis, resulting in a sample of 185 patients used for the per-protocol analysis. *Two allocation errors **one protocol violation were already lost to follow-up – those wore not subtracted at this stage.

2. *Table S1: Excluded Patients from Study Participation*

	N
**Not meeting inclusion criteria**	
Primary complaint tinnitus	2
Chronic tinnitus (≥ six months)	1
Age between 18 and 80 years	2
A score of ≥ 18 in the Tinnitus Handicap Inventory (THI; Newman et al., 1996) – at least mild tinnitus distress	32
A score of > 22 in the Montreal Cognitive Assessment (MoCa; Nasreddine et al., 2005) – absence of mild cognitive impairment	14
Ability and willingness to use the UNITI mobile applications on smartphones	4
Openness to use a HA (if indication and allocation to HA group)	..
Ability to understand and consent to the research (hearing ability, intellectual capacity)	..
Ability to participate in all relevant visits (no plans for e.g., long-term holidays or pregnancy)	..
Existing drug therapies with psychoactive substances (e.g., antidepressants or anticonvulsants) must be stable for at least 30 days at the beginning of the therapeutic intervention. The drug therapy should remain constant during the course of the study. Necessary changes do not constitute an exclusion criterion per se, but need to be recorded.	..
**Meeting exclusion criteria**	
Objective tinnitus or heartbeat-synchronous tinnitus as primary complaint	7
Otosclerosis / acoustic neuroma or other relevant ear disorders with fluctuation hearing	10
Present acute infections (acute otitis media, otitis externa, acute sinusitis)	1
Meniere's disease or similar syndromes (but not vestibular migraine)	2
Serious internal, neurological or psychiatric conditions	4
Epilepsy or other disorders of the central nervous system (e.g., brain tumor or encephalitis)	1
Clinically relevant drug, medication or alcohol abuse up to 12 weeks before study start	..
Severe hearing loss – inability to communicate properly in the course of the study	11
One deaf ear	4
Missing written informed consent	1
Start of any other tinnitus related treatments, especially hearing aids, structured counselling, sound therapy (with special devices; expecting long term effects) or cognitive behavioural therapy in the last 3 months before the start of the study	3
**Other reasons**	
Stratification group completed	107
Personal reasons/ declined to participate	6
Artificial eardrum	1
**Total number of excluded patients**	213

Note. We aimed for four equally sized stratification groups of 25 patients per clinical site. 107 patients could not be included in the trial since the respective stratification group (e.g., high tinnitus-related handicap and no hearing aid indication) was already full.

3. *Table S2: Quantity and Reasons of Dropouts before Treatment Start per Treatment Arm*

Treatment arm	N	Reasons (N)
CBT	9	No time (2); unable to travel to clinic (2); rejected CBT (2); no internet connection (1); started another treatment (1); interested in HA (1)
HA	1	Declined to participate (1)
SC	4	Rejected SC (1); interested in HA (1); declined to participate (1); app treatment not started (1)
ST	1	No capacity for daily app usage (1)
CBT + HA	5	Unable to travel to clinic (1); rejected HA and CBT (1); No capacity for CBT (1); suspected Morbus Meniere (1); lost (1)
CBT + SC	11	No time (4); lost (2); financial reasons (1); job reasons (1); chronic fatigue syndrome (1); rejected CBT (1); rejected CBT & app treatment not started (1)
CBT + ST	9	Rejected CBT + ST (3); no time (3); lost (1); personal reasons (1); job reasons (1)
HA + SC	1	Unexpected personal problems (1)
HA + ST	1	Rejected HA (1)
SC + ST	4	Interested in HA (1); no time (1); suspected acoustic neuroma (1); app treatment not started (1)
Total number of dropouts before treatment start	46	

Note. CBT = Cognitive-Behavioural Therapy; HA = Hearing Aids; SC = Structured Counselling; ST = Sound Therapy.

4. *Table S3: Quantity and Reasons of Dropouts during Treatment per Treatment Arm – before Interim Visit*

**Treatment arm**	**N**	**Reasons (N)**
CBT	4	Issues with daily app measures (1); no time (1); health worsening (1); personal reasons (1)
HA	1	Rejected HA (1)
SC	2	Not satisfied with treatment (1); lack of benefit & issues with daily app measures (1)
ST	3	Tinnitus worsening (1); tinnitus worsening & not satisfied with app treatment; no time due to illness (1)
CBT + HA	..	..
CBT + SC	6	Unsatisfied with CBT (1); health reasons (1); lost (1); no benefit of CBT (1); job reasons (1); rejected CBT (1)
CBT + ST	6	Tinnitus worsening (1); lost (1); no time (1); unable to travel to clinic (1); rejected CBT (1); treatment termination after first CBT session (1)
HA + SC	..	..
HA + ST	..	..
SC + ST	3	Tinnitus worsening (1); no reasons (1); lost (1)
Total number of dropouts during treatment – before interim visit	25	

Note. CBT = Cognitive-Behavioural Therapy; HA = Hearing Aids; SC = Structured Counselling; ST = Sound Therapy.

5. ***Table S4: Quantity and Reasons of Dropouts during Treatment per Treatment Arm – after Interim Visit***

**Treatment arm**	**N**	**Reasons (N)**
CBT	4	No time (2); unable to travel to clinic (1); job reasons (1)
HA	3	Lost (2); started another HA treatment (1)
SC	5	Tinnitus worsening (1); lost (1); lack of benefit (1); health worsening/ reasons (1); did not want to continue (1)
ST	3	Not satisfied with treatment (2); lost (1)
CBT + HA	1	No time (1)
CBT + SC	2	No time (1); no longer attends CBT sessions (1)
CBT + ST	4	No benefit of CBT (2); tinnitus worsening (1); lost (1)
HA + SC	..	..
HA + ST	1	App treatment was never started (1)
SC + ST	3	App treatment was never started (1); lost (1); did not want to come to clinic due to COVID-19 pandemic (1)
Total number of dropouts during treatment – after interim visit	26	

Note. CBT = Cognitive-Behavioural Therapy; HA = Hearing Aids; SC = Structured Counselling; ST = Sound Therapy.

6. Table S5: Quantity and Reasons of Patients lost to Follow-up

**Treatment arm**	**N**	**Reasons (N)**
CBT	5	No response (3); health reasons (1); no reasons (1)
HA	5	Job reasons (2); no response (2); no reasons (1)
SC	11	No response (6); not interested in follow-up (3); no reasons (1); health reasons (1)
ST	2	No response (1); not available for follow-up (1)
CBT + HA	1	No response (1)
CBT + SC	3	No response (1); not interested in follow-up (1); terminated follow-up too late (1)
CBT + ST	1	Deceased (1)
HA + SC	1	No response (1)
HA + ST	3	No response (3)
SC + ST	4	No response (2); wanted to discontinue (1); not available for follow-up (1)
Total number of patients lost to follow-up	36	

Note. CBT = Cognitive-Behavioural Therapy; HA = Hearing Aids; SC = Structured Counselling; ST = Sound Therapy.

7. *Table S6: Demographic and Clinical Characteristics of the Participants at Baseline (stratified by Treatment Arm).*

	**CBT (n=56)**	**HA (n=59)**	**SC (n=56)**	**ST (n=59)**	**CBT + HA (n=17)**	**CBT + SC (n=51)**	**CBT + ST (n=54)**	**HA + SC (N=19)**	**HA + ST (n=27)**	**SC + ST (n=63)**	**Total (N=461)**
**Education — no. (%)**											
Elementary or middle school	11 (21.2%)	18 (30.5%)	12 (21.8%)	12 (21.4%)	7 (43.8%)	20 (41.7%)	10 (20.0%)	4 (21.1%)	6 (22.2%)	17 (27.0%)	117 (26.3%)
High school	10 (19.2%)	10 (16.9%)	12 (21.8%)	16 (28.6%)	2 (12.5%)	7 (14.6%)	11 (22.0%)	6 (31.6%)	6 (22.2%)	13 (20.6%)	93 (20.9%)
University	31 (59.6%)	31 (52.5%)	31 (56.4%)	28 (50.0%)	7 (43.8%)	21 (43.8%)	29 (58.0%)	9 (47.4%)	15 (55.6%)	33 (52.4%)	235 (52.8%)
**Tinnitus Presentation (ESIT-SQ B2) — no. (%)**											
constant	48 (94.1%)	57 (96.6%)	49 (92.5%)	51 (94.4%)	13 (86.7%)	44 (93.6%)	45 (93.8%)	18 (100%)	26 (100%)	57 (90.5%)	408 (94.0%)
intermittent	3 (5.88%)	2 (3.39%)	4 (7.55%)	3 (5.56%)	2 (13.3%)	3 (6.38%)	3 (6.25%)	0 (0%)	0 (0%)	6 (9.52%)	26 (5.99%)
**Tinnitus Loudness [dB] (Tinnitus Matching)**											
Mean (SD)	35.3 (21.6)	42.0 (21.1)	32.8 (23.0)	37.4 (23.1)	48.7 (18.6)	37.9 (19.1)	37.4 (18.6)	44.9 (27.6)	51.4 (19.9)	38.0 (21.8)	38.8 (21.7)
Median [Min, Max]	37.3 [1, 83]	46.0 [0, 82.5]	30.0 [2.5, 88]	35.0 [5, 90]	48.0 [20, 78]	40.5 [0, 75]	35.0 [5, 85]	41.3 [0, 100]	53.8 [10, 95]	35.0 [2.50, 92]	40.0 [0, 100]
**Residual inhibition: Both ears — no. (%)**											
none	17 (70.8%)	10 (41.7%)	11 (45.8%)	16 (50.0%)	3 (42.9%)	14 (56.0%)	17 (53.1%)	5 (50.0%)	8 (57.1%)	17 (48.6%)	118 (52.0%)
partially	5 (20.8%)	10 (41.7%)	8 (33.3%)	8 (25.0%)	2 (28.6%)	5 (20.0%)	7 (21.9%)	4 (40.0%)	5 (35.7%)	7 (20.0%)	61 (26.9%)
complete	2 (8.33%)	4 (16.7%)	5 (20.8%)	8 (25.0%)	2 (28.6%)	6 (24.0%)	8 (25.0%)	1 (10.0%)	1 (7.14%)	11 (31.4%)	48 (21.1%)
**Residual inhibition: Left ear — no. (%)**											
none	9 (50.0%)	10 (62.5%)	9 (56.3%)	10 (71.4%)	2 (66.7%)	7 (63.6%)	7 (70.0%)	3 (75.0%)	1 (16.7%)	4 (33.3%)	62 (56.4%)
partially	5 (27.8%)	2 (12.5%)	1 (6.25%)	2 (14.3%)	0 (0%)	2 (18.2%)	1 (10.0%)	1 (25.0%)	3 (50.0%)	6 (50.0%)	23 (20.9%)

	CBT (n=56)	HA (n=59)	SC (n=56)	ST (n=59)	CBT + HA (n=17)	CBT + SC (n=51)	CBT + ST (n=54)	HA + SC (N=19)	HA + ST (n=27)	SC + ST (n=63)	Total (N=461)
complete	4 (22.2%)	4 (25.0%)	6 (37.5%)	2 (14.3%)	1 (33.3%)	2 (18.2%)	2 (20.0%)	0 (0%)	2 (33.3%)	2 (16.7%)	25 (22.7%)
**Residual inhibition: Right ear — no. (%)**											
none	9 (60.0%)	12 (63.2%)	11 (78.6%)	10 (90.9%)	5 (83.3%)	10 (66.7%)	6 (75.0%)	3 (60.0%)	3 (60.0%)	6 (54.5%)	75 (68.8%)
partially	2 (13.3%)	4 (21.1%)	1 (7.14%)	0 (0%)	0 (0%)	1 (6.67%)	1 (12.5%)	2 (40.0%)	0 (0%)	3 (27.3%)	14 (12.8%)
complete	4 (26.7%)	3 (15.8%)	2 (14.3%)	1 (9.09%)	1 (16.7%)	4 (26.7%)	1 (12.5%)	0 (0%)	2 (40.0%)	2 (18.2%)	20 (18.3%)
**WHO-QoL 1 score: Physical health**											
Mean (SD)	12.4 (1.73)	12.5 (1.78)	12.4 (1.84)	11.8 (2.10)	13.0 (1.62)	12.9 (2.04)	12.6 (1.57)	13.1 (1.26)	12.7 (2.11)	12.7 (1.96)	12.5 (1.87)
Median [Min, Max]	13.0 [7, 15]	13.0 [7, 15]	13.0 [7, 17]	11.5 [7, 16]	13.0 [10, 16]	13.0 [9, 17]	13.0 [10, 15]	13.0 [11, 15]	13.0 [10, 17]	13.0 [8, 17]	13.0 [7, 17]
**WHO-QoL 2 score: Psychological health**											
Mean (SD)	13.3 (1.92)	13.6 (1.61)	13.5 (1.99)	13.2 (2.02)	13.9 (1.03)	14.1 (1.84)	13.7 (1.72)	14.2 (1.48)	13.3 (2.09)	13.5 (1.95)	13.6 (1.85)
Median [Min, Max]	13.0 [8, 17]	14.0 [9, 16]	14.0 [9, 18]	13.0 [6, 17]	14.0 [12, 16]	14.0 [10, 17]	14.0 [11, 17]	14.0 [11, 17]	13.0 [8, 17]	14.0 [9, 17]	14.0 [6, 18]
**WHO-QoL 3 score: Social factors**											
Mean (SD)	14.5 (3.27)	14.3 (3.15)	14.5 (3.03)	14.0 (3.11)	14.6 (2.83)	14.8 (3.01)	14.6 (3.15)	15.1 (2.78)	14.6 (3.14)	14.9 (2.96)	14.5 (3.06)
Median [Min, Max]	15.0 [8, 20]	15.0 [5, 20]	15.0 [8, 20]	15.0 [4, 20]	15.0 [9, 20]	15.0 [8, 20]	15.0 [9, 20]	15.5 [9, 20]	15.0 [8, 20]	16.0 [7, 20]	15.0 [4, 20]
**WHO-QoL 4 score: Environment**											
Mean (SD)	15.8 (2.09)	15.9 (2.15)	15.7 (2.59)	15.5 (2.35)	16.1 (1.82)	16.4 (1.93)	15.6 (1.92)	15.8 (1.77)	15.7 (2.38)	16.0 (2.30)	15.8 (2.18)
Median [Min, Max]	16.0 [11, 20]	16.0 [8, 20]	16.0 [9, 20]	15.5 [9, 20]	16.0 [13, 19]	16.0 [13, 20]	16.0 [12, 20]	15.5 [13, 19]	16.0 [11, 20]	16.0 [12, 20]	16.0 [8, 20]

Note. Data are n (%), mean (SD), or median [Min, Max]. Tinnitus loudness level in dB was obtained by tinnitus matching, with higher values indicating greater perceived tinnitus loudness. Residual inhibition is the brief tinnitus suppression following acoustic stimulation. WHO-QoL scores range from 4 to 20, with higher scores indicating higher quality of life in the respective domain. CBT = Cognitive-Behavioural Therapy; HA = Hearing Aids; SC = Structured Counselling; ST = Sound Therapy.

8. *Figure S2: Audiogram in Patients with and Without Hearing Aid Indication for Single and Combined Treatment Groups*

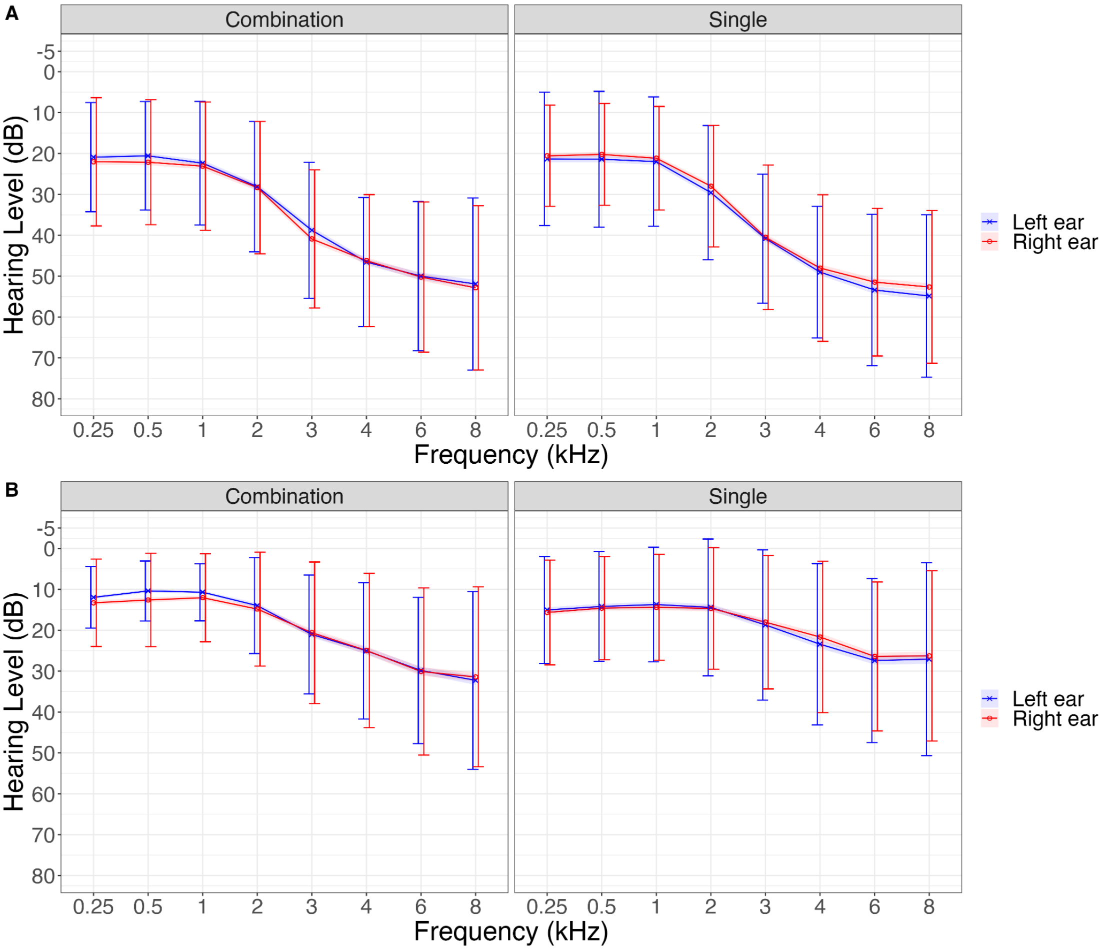

Note. Pooled audiogram for patients (A) with hearing aid indication and (B) without hearing aid indication separated for single and combined treatment groups measured by pure tone audiometry at baseline visit according to the guidelines of the British Society of Audiology. Error bars represent standard deviation.

9. Figure S3: Audiogram for Patients in all Treatment Groups

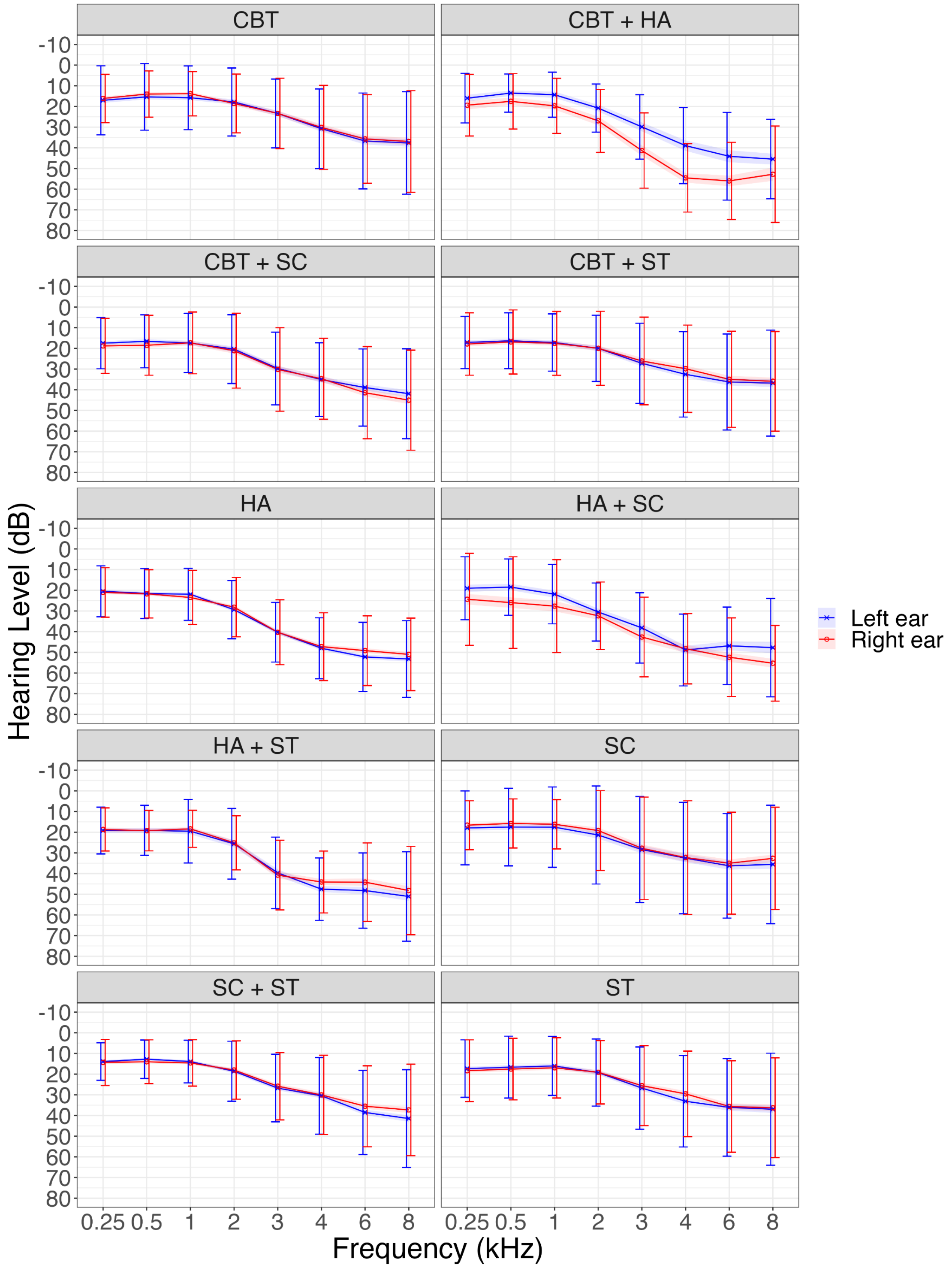

Note. Pooled audiograms of patients in all treatment groups measured by pure tone audiometry at baseline visit according to the guidelines of the British Society of Audiology. Error bars represent standard deviation. CBT = Cognitive-Behavioural Therapy; HA = Hearing Aids; SC = Structured Counselling; ST = Sound Therapy.

10. Table S7: Representativeness of Study Participants

**Category**	**Example**
Condition under investigation	Chronic tinnitus. The Tinnitus persisted for a minimum of 6 months.
Sex	No sex effect was found for the primary outcome (THI) when comparing single versus combination treatments for chronic tinnitus. 41.2 % of the participants were female, 58.8% were male.
Age	No age effect was found for the primary outcome (THI) when comparing single versus combination treatments for chronic tinnitus.<br><br>The average age was 51.1 years. In a study by Probst et al. 2017, it was reported that the majority of patients visiting the specialised tinnitus clinic in Regensburg are in the age range of 45 - 55 years.
Race or ethnic group	Race or ethnicity was not assessed. No conclusions can be drawn about the influence of race or ethnicity.
Geography	The 5 centres were located in four European countries: Germany (2), Greece, Netherlands, Spain. Conclusions about countries with dissimilar health care systems are limited.
Severity	The average THI score at baseline was 48.0, which is considered moderate tinnitus distress. Participants with slight or no distress at screening (THI < 18), were excluded from the study.
Other considerations	In this trial, we compared single versus combination treatment of four clinical intervention types typically used for tinnitus treatment: cognitive behavioural therapy, hearing aids, sound therapy, and structured counselling for a duration of 12 weeks. The treatments were implemented in a typical way. Conclusions about treatments with different implementations are limited.
Overall representativeness of this trial	This trial showed that combining a treatment of weak clinical efficacy with a treatment of stronger clinical efficacy leads to an improvement of the overall clinical outcome.<br><br>We recognise that the trial was limited to only four different clinical intervention types and their combinations. We do not know whether other treatment combinations, which are not part of the trial, would be superior to their respective singe treatments.<br><br>We cannot conclude about combination treatments with three or more clinical interventions.<br><br>All treatments were implemented in a typical way; however, it would be possible to implement them differently or apply the treatment for a shorter or longer time. We do not know whether other implementation would have the same results.

11. *Table S8: Primary Outcome (THI) Objective 1: Model parameters*

Characteristic	Beta	95% CI[1]	p-value
visit_type			
baseline	—	—	
interim_visit	-10.497	-12.622, -8.373	**<0.001**
final_visit	-14.864	-16.998, -12.730	**<0.001**
followup_1	-13.306	-15.522, -11.090	**<0.001**
objective_1			
Combination	—	—	
Single	-0.178	-3.293, 2.938	0.911
sex			
female	—	—	
male	-0.891	-3.690, 1.908	0.532
age_baseline	-0.034	-0.154, 0.086	0.575
ha_indication			
ha	—	—	
no_ha	-3.509	-6.367, -0.650	**0.016**
phq9_score_baseline	2.132	1.852, 2.411	**<0.001**
esitsq_a5_education_baseline			
elementary_middle	—	—	
high_school	1.949	-2.182, 6.079	0.355
university	-3.428	-6.921, 0.065	0.054
visit_type * objective_1			
interim_visit * Single	2.647	-0.376, 5.669	0.086
final_visit * Single	3.159	0.235, 6.083	**0.034**
followup_1 * Single	1.382	-1.632, 4.396	0.368

[1] CI = Confidence Interval

Note. The table depicts the parameters of the linear mixed effects model (using REML in the lme4 R package) predicting the primary outcome by objective, time point (baseline, interim visit, final visit, and follow-up), and objective-by-time interaction as fixed effects, including centre and subject ID as random intercepts. The model was adjusted for the following covariates: age, sex, educational attainment, hearing aid indication, and PHQ-9 baseline scores. All comparisons are two-sided and p values reported here were not adjusted for multiple comparisons.

12. *Figure S4: Primary Outcome (THI) Objective 1: Model Assumptions*

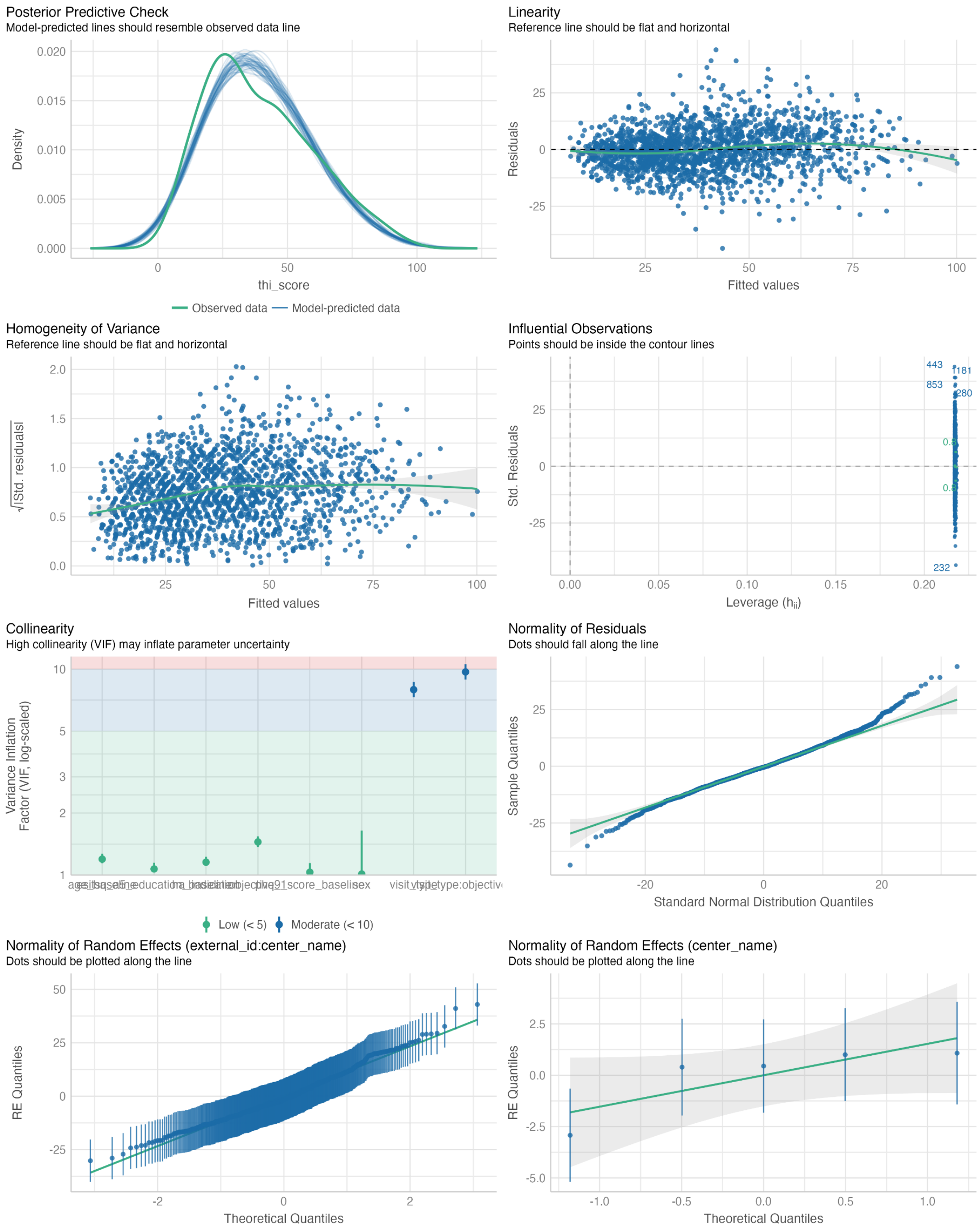

Note. Model assumptions are plotted for the first of the 50 imputed datasets.

13. *Figure S5: Primary Outcome (THI) Objective 2*

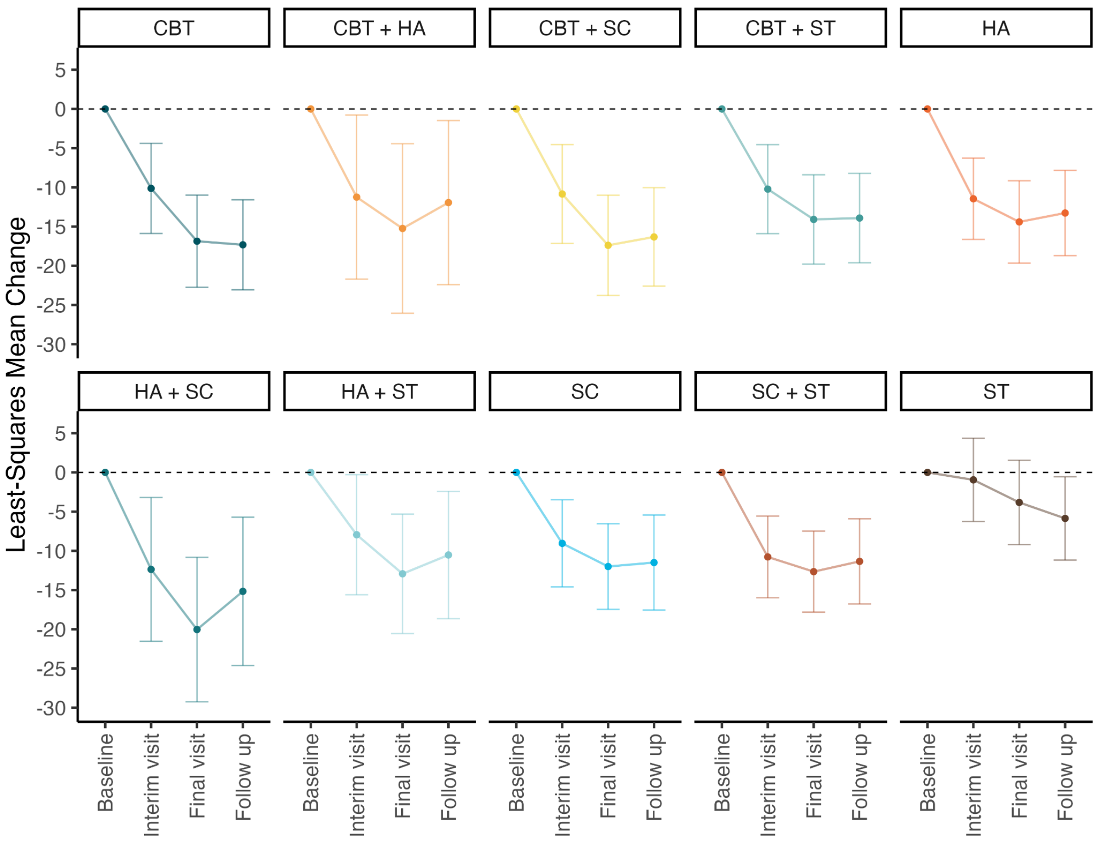

Note. Total THI scores range from 0 to 100, with higher scores indicating greater severity of tinnitus. Error bars represent 95% confidence intervals. Sample sizes: CBT: N = 56, HA: N = 59, SC: N = 56, ST: N = 59, CBT + HA: N = 17, CBT + SC: N = 51, CBT + ST: N = 54, HA + SC: N = 19, HA + ST: N = 27, SC + ST: N = 63. CBT = Cognitive-Behavioural Therapy; HA = Hearing Aids; SC = Structured Counselling; ST = Sound Therapy.

14. Table S9: THI Change from Baseline to Final Visit for Hearing Aid Indication (yes/no)

Contrast	Change from baseline (Hearing aid indication: yes)	Change from baseline (Hearing aid indication: no)
CBT	-10.4 [-20.9, 0]	-20.2 [-27.1, -13.2]
CBT + HA	-15.2 [-26, -4.5]	NA
CBT + SC	-18.6 [-28.3, -8.9]	-16.7 [-25.1, -8.4]
CBT + ST	-11.4 [-21.3, -1.6]	-15.3 [-22.4, -8.2]
HA	-14.4 [-19.6, -9.2]	NA
HA + SC	-20 [-29.2, -10.9]	NA
HA + ST	-12.9 [-20.5, -5.4]	NA
SC	-12.8 [-22.5, -3.1]	-11.6 [-18.3, -4.8]
SC + ST	-13.1 [-22.4, -3.9]	-12.5 [-18.7, -6.3]
ST	-3 [-12, 6.1]	-4.3 [-10.9, 2.4]

Note. Values depict least-squares mean changes with 95% CI in square brackets. CBT = Cognitive-Behavioural Therapy; HA = Hearing Aids; SC = Structured Counselling; ST = Sound Therapy.

15. Table S10: THI Change from Baseline to Final Visit for High and Low Tinnitus Distress Severity

**Contrast**	**Change from baseline (High tinnitus distress severity)**	**Change from baseline (Low tinnitus distress severity)**
CBT	-25 [-34.2, -15.8]	-9.3 [-16, -2.5]
CBT + HA	-18.7 [-36.6, -0.8]	-12.2 [-24, -0.3]
CBT + SC	-24.4 [-33.7, -15.1]	-10.6 [-18.6, -2.6]
CBT + ST	-23.2 [-32.2, -14.3]	-6.2 [-13.1, 0.7]
HA	-18.1 [-26.1, -10.1]	-10.6 [-17.2, -4.1]
HA + SC	-26.9 [-41.6, -12.2]	-13.8 [-24.5, -3.2]
HA + ST	-12.8 [-24.4, -1.2]	-13.1 [-22.4, -3.8]
SC	-15.6 [-24.6, -6.7]	-8.8 [-15.2, -2.5]
SC + ST	-19.1 [-27.8, -10.5]	-7.8 [-13.5, -2.1]
ST	-6.3 [-14.6, 2.1]	-1.5 [-7.8, 4.8]

Note. Values depict least-squares mean changes with 95% CI in square brackets. High tinnitus distress severity: THI $\geq$ 48 at Screening. Low tinnitus distress severity: THI < 48 at Screening. CBT = Cognitive-Behavioural Therapy; HA = Hearing Aids; SC = Structured Counselling; ST = Sound Therapy.

16. *Table S11: Primary Outcome (THI) Objective 4 & 5 at Final Visit*

Objective	Contrast	Change from baseline	95% CI
4_A	Doesnt_have_CBT	-11.57	[-14.01, -9.14]
4_A	Has_CBT	-16.02	[-19.33, -12.70]
4_B	Doesnt_have_HA	-11.42	[-15.47, -7.38]
4_B	Has_HA	-15.07	[-18.75, -11.39]
4_C	Doesnt_have_SC	-12.46	[-15.01, -9.91]
4_C	Has_SC	-14.48	[-17.62, -11.34]
4_D	Doesnt_have_ST	-15.48	[-18.23, -12.72]
4_D	Has_ST	-10.51	[-13.43, -7.58]
5	Brain	-15.36	[-18.82, -11.89]
5	Brain_and_Ear	-14.36	[-17.69, -11.04]
5	Ear	-9.83	[-13.20, -6.45]

Note. Values depict least-squares mean changes with 95% CI in square brackets. Objective 4: Treatments with vs. treatments without; Objective 5: Ear mediated vs. brain mediated vs. ear and brain mediated treatments. CBT = Cognitive-Behavioural Therapy; HA = Hearing Aids; SC = Structured Counselling; ST = Sound Therapy.

17. Table S12: Primary Outcome (THI) at Interim Visit

Objective	Contrast	Change from baseline	95% CI
1	Combination	-10.50	[-13.28, -7.71]
1	Single	-7.85	[-10.66, -5.04]
2	CBT	-10.13	[-15.87, -4.38]
2	CBT + HA	-11.24	[-21.70, -0.78]
2	CBT + SC	-10.84	[-17.15, -4.54]
2	CBT + ST	-10.22	[-15.90, -4.55]
2	HA	-11.45	[-16.63, -6.27]
2	HA + SC	-12.37	[-21.53, -3.21]
2	HA + ST	-7.95	[-15.61, -0.29]
2	SC	-9.05	[-14.60, -3.50]
2	SC + ST	-10.78	[-15.99, -5.57]
2	ST	-0.95	[-6.25, 4.35]
3_A	Combi_CBT	-10.62	[-14.57, -6.68]
3_A	Single_CBT	-10.13	[-15.92, -4.33]
3_B	Combi_HA	-10.17	[-15.05, -5.28]
3_B	Single_HA	-11.45	[-16.42, -6.48]
3_C	Combi_SC	-11.03	[-14.72, -7.34]
3_C	Single_SC	-9.05	[-14.60, -3.50]
3_D	Combi_ST	-10.04	[-13.57, -6.51]
3_D	Single_ST	-0.95	[-6.35, 4.46]
4_A	Doesnt_have_CBT	-8.36	[-10.83, -5.90]
4_A	Has_CBT	-10.47	[-13.70, -7.23]
4_B	Doesnt_have_HA	-7.77	[-11.79, -3.76]
4_B	Has_HA	-10.79	[-14.39, -7.19]
4_C	Doesnt_have_SC	-8.30	[-10.87, -5.72]
4_C	Has_SC	-10.44	[-13.54, -7.35]
4_D	Doesnt_have_ST	-10.58	[-13.23, -7.92]
4_D	Has_ST	-7.40	[-10.34, -4.45]
5	Brain	-9.98	[-13.33, -6.63]
5	Brain_and_Ear	-10.83	[-14.19, -7.47]
5	Ear	-6.53	[-9.91, -3.14]

Note. Values depict least-squares mean changes with 95% CI in square brackets. Objective 1: Single vs. Combination (all treatments); Objective 2: All treatments against each other; Objective 3: Single vs. Combination (separately); Objective 4: Treatments with vs. treatments without; Objective 5: Ear-mediated vs. brain-mediated vs. ear- and brain-mediated treatments. CBT = Cognitive-Behavioural Therapy; HA = Hearing Aids; SC = Structured Counselling; ST = Sound Therapy.

18. Table S13: Primary Outcome (THI) at Follow-up

Objective	Contrast	Change from baseline	95% CI
1	Combination	-13.31	[-16.21, -10.40]
1	Single	-11.92	[-14.75, -9.09]
2	CBT	-17.32	[-23.06, -11.58]
2	CBT + HA	-11.94	[-22.40, -1.48]
2	CBT + SC	-16.32	[-22.60, -10.04]
2	CBT + ST	-13.91	[-19.62, -8.20]
2	HA	-13.27	[-18.70, -7.83]
2	HA + SC	-15.17	[-24.62, -5.71]
2	HA + ST	-10.53	[-18.65, -2.41]
2	SC	-11.50	[-17.55, -5.44]
2	SC + ST	-11.35	[-16.77, -5.92]
2	ST	-5.87	[-11.18, -0.55]
3_A	Combi_CBT	-14.64	[-18.56, -10.72]
3_A	Single_CBT	-17.32	[-23.11, -11.53]
3_B	Combi_HA	-12.31	[-17.40, -7.21]
3_B	Single_HA	-13.27	[-18.51, -8.03]
3_C	Combi_SC	-13.80	[-17.67, -9.93]
3_C	Single_SC	-11.50	[-17.55, -5.44]
3_D	Combi_ST	-12.15	[-15.79, -8.52]
3_D	Single_ST	-5.87	[-11.28, -0.45]
4_A	Doesnt_have_CBT	-10.81	[-13.39, -8.23]
4_A	Has_CBT	-15.49	[-18.75, -12.22]
4_B	Doesnt_have_HA	-11.46	[-15.62, -7.29]
4_B	Has_HA	-12.77	[-16.59, -8.95]
4_C	Doesnt_have_SC	-12.27	[-14.90, -9.64]
4_C	Has_SC	-13.12	[-16.48, -9.75]
4_D	Doesnt_have_ST	-14.42	[-17.15, -11.69]
4_D	Has_ST	-10.33	[-13.30, -7.36]
5	Brain	-15.01	[-18.56, -11.45]
5	Brain_and_Ear	-12.79	[-16.23, -9.35]
5	Ear	-9.75	[-13.22, -6.28]

Note. Values depict least-squares mean changes with 95% CI in square brackets. Objective 1: Single vs. Combination (all treatments); Objective 2: All treatments against each other; Objective 3: Single vs. Combination (separately); Objective 4: Treatments with vs. treatments without; Objective 5: Ear-mediated vs. brain-mediated vs. ear- and brain-mediated treatments. CBT = Cognitive-Behavioural Therapy; HA = Hearing Aids; SC = Structured Counselling; ST = Sound Therapy.

19. Table S14: THI Change from Baseline to Final Visit for each Country

**Contrast**	**Belgium**	**Germany**	**Greece**	**Spain**
Single	-9.4 [-3.2; -15.5]	-12.0 [-8.0; -16.0]	-16.0 [-9.2; -22.8]	-8.8 [-2.4; -15.2]
Combination	-13.1 [-4.9; -21.2]	-16.5 [-12.6; -20.4]	-15.8 [-9.3; -22.3]	-11.3 [-5.1; -17.5]
CBT	-19.1 [-5.7; -32.4]	-16.4 [-8.0; -24.8]	-20.4 [-6.8; -34.0]	-11.4 [2.6; -25.5]
CBT + HA	-38.0 [0.7; -76.7]	-14.1 [0.8; -29.1]	-11.1 [14.8; -37.0]	-16.0 [5.7; -37.6]
CBT + SC	-13.7 [6.5; -33.9]	-21.1 [-12.8; -29.4]	-15.7 [-2.0; -29.5]	-12.2 [2.8; -27.3]
CBT + ST	-12.9 [5.8; -31.6]	-17.5 [-10.0; -25.0]	-11.8 [2.2; -25.7]	-10.1 [2.3; -22.6]
HA	-12.6 [-1.1; -24.2]	-15.5 [-8.2; -22.8]	-21.2 [-7.5; -34.8]	-7.8 [5.0; -20.6]
HA + SC	-26.7 [-2.0; -51.5]	-19.5 [-6.8; -32.2]	-21.1 [2.3; -44.6]	-15.0 [4.6; -34.6]
HA + ST	4.7 [27.0; -17.7]	-9.9 [1.0; -20.9]	-30.3 [-12.6; -48.1]	-10.3 [5.6; -26.3]
SC	-7.1 [5.3; -19.5]	-12.7 [-4.8; -20.6]	-13.5 [0.3; -27.3]	-14.9 [-1.2; -28.5]
SC + ST	-11.5 [1.1; -24.1]	-14.2 [-6.9; -21.5]	-13.0 [-0.6; -25.5]	-9.3 [3.5; -22.0]
ST	-0.3 [11.5; -12.1]	-3.4 [4.0; -10.9]	-9.3 [4.1; -22.6]	-2.6 [10.4; -15.6]

Note. Values depict least-squares mean changes with 95% CI in square brackets. Comparison of single and combination treatments as well as all treatments regarding the THI difference from baseline to final visit for each country. Note that there were two centers in Germany (Berlin, Regensburg).

20. Table S15: Secondary Outcome (TFI) Objective 4 & 5 at Final Visit

Objective	Contrast	Change from baseline	95% CI
4_A	Doesnt_have_CBT	-10.01	[-12.66, -7.36]
4_A	Has_CBT	-13.33	[-16.89, -9.78]
4_B	Doesnt_have_HA	-9.25	[-13.64, -4.86]
4_B	Has_HA	-14.19	[-18.09, -10.29]
4_C	Doesnt_have_SC	-12.21	[-14.96, -9.46]
4_C	Has_SC	-9.97	[-13.33, -6.61]
4_D	Doesnt_have_ST	-12.80	[-15.62, -9.99]
4_D	Has_ST	-9.37	[-12.56, -6.18]
5	Brain	-12.26	[-15.89, -8.64]
5	Brain_and_Ear	-11.13	[-14.86, -7.40]
5	Ear	-10.37	[-14.00, -6.73]

Note. Values depict least-squares mean changes with 95% CI in square brackets. Objective 4: Treatments with vs. treatments without; Objective 5: Ear-mediated vs. brain-mediated vs. ear- and brain-mediated treatments. CBT = Cognitive-Behavioural Therapy; HA = Hearing Aids; SC = Structured Counselling; ST = Sound Therapy.

21. *Table S16: Secondary Outcome (Mini-TQ) Objective 4 & 5 at Final Visit*

Objective	Contrast	Change from baseline	95% CI
4_A	Doesnt_have_CBT	-2.63	[-3.25, -2.02]
4_A	Has_CBT	-3.91	[-4.71, -3.10]
4_B	Doesnt_have_HA	-2.51	[-3.49, -1.53]
4_B	Has_HA	-3.28	[-4.16, -2.41]
4_C	Doesnt_have_SC	-3.05	[-3.69, -2.41]
4_C	Has_SC	-3.23	[-3.99, -2.48]
4_D	Doesnt_have_ST	-3.61	[-4.27, -2.95]
4_D	Has_ST	-2.50	[-3.24, -1.77]
5	Brain	-3.65	[-4.50, -2.80]
5	Brain_and_Ear	-3.30	[-4.15, -2.44]
5	Ear	-2.35	[-3.21, -1.50]

Note. Values depict least-squares mean changes with 95% CI in square brackets. Objective 4: Treatments with vs. treatments without; Objective 5: Ear-mediated vs. brain-mediated vs. ear- and brain-mediated treatments. CBT = Cognitive-Behavioural Therapy; HA = Hearing Aids; SC = Structured Counselling; ST = Sound Therapy.

22. *Table S17: Secondary Outcome (NRS-2: "How strong or loud is your tinnitus at present?") Objective 4 & 5 at Final Visit*

Objective	Contrast	Change from baseline	95% CI
4_A	Doesnt_have_CBT	-0.80	[-1.15, -0.45]
4_A	Has_CBT	-0.73	[-1.21, -0.25]
4_B	Doesnt_have_HA	-0.44	[-1.03, 0.15]
4_B	Has_HA	-1.10	[-1.63, -0.57]
4_C	Doesnt_have_SC	-0.79	[-1.15, -0.42]
4_C	Has_SC	-0.76	[-1.20, -0.31]
4_D	Doesnt_have_ST	-0.87	[-1.27, -0.48]
4_D	Has_ST	-0.64	[-1.05, -0.24]
5	Brain	-0.75	[-1.26, -0.24]
5	Brain_and_Ear	-0.68	[-1.17, -0.19]
5	Ear	-0.90	[-1.38, -0.42]

Note. Values depict least-squares mean changes with 95% CI in square brackets. Objective 4: Treatments with vs. treatments without; Objective 5: Ear-mediated vs. brain-mediated vs. ear- and brain-mediated treatments. CBT = Cognitive-Behavioural Therapy; HA = Hearing Aids; SC = Structured Counselling; ST = Sound Therapy.

23. *Table S18: Secondary Outcome (PHQ-9) Objective 4 & 5 at Final Visit*

Objective	Contrast	Change from baseline	95% CI
4_A	Doesnt_have_CBT	-1.46	[-2.04, -0.88]
4_A	Has_CBT	-1.70	[-2.45, -0.95]
4_B	Doesnt_have_HA	-1.28	[-2.25, -0.32]
4_B	Has_HA	-1.90	[-2.76, -1.05]
4_C	Doesnt_have_SC	-1.62	[-2.22, -1.02]
4_C	Has_SC	-1.46	[-2.20, -0.73]
4_D	Doesnt_have_ST	-1.85	[-2.48, -1.23]
4_D	Has_ST	-1.17	[-1.87, -0.48]
5	Brain	-1.72	[-2.52, -0.93]
5	Brain_and_Ear	-1.36	[-2.17, -0.55]
5	Ear	-1.57	[-2.37, -0.77]

Note. Values depict least-squares mean changes with 95% CI in square brackets. Objective 4: Treatments with vs. treatments without; Objective 5: Ear-mediated vs. brain-mediated vs. ear- and brain-mediated treatments. CBT = Cognitive-Behavioural Therapy; HA = Hearing Aids; SC = Structured Counselling; ST = Sound Therapy.

24. *Table S19: Secondary Outcome (WHO-QoL-Bref 1: Physical Health) at Final Visit*

Objective	Contrast	Change from baseline	95% CI
1	Combination	0.29	[0.01, 0.58]
1	Single	0.42	[0.13, 0.71]
2	CBT	0.43	[-0.18, 1.04]
2	CBT + HA	0.49	[-0.64, 1.62]
2	CBT + SC	0.24	[-0.39, 0.88]
2	CBT + ST	0.36	[-0.23, 0.95]
2	HA	0.52	[-0.01, 1.06]
2	HA + SC	0.64	[-0.32, 1.60]
2	HA + ST	0.36	[-0.43, 1.15]
2	SC	0.20	[-0.39, 0.79]
2	SC + ST	0.10	[-0.44, 0.63]
2	ST	0.51	[-0.04, 1.07]
3_A	Combi_CBT	0.33	[-0.07, 0.73]
3_A	Single_CBT	0.43	[-0.17, 1.03]
3_B	Combi_HA	0.48	[-0.06, 1.01]
3_B	Single_HA	0.52	[-0.01, 1.06]
3_C	Combi_SC	0.23	[-0.15, 0.60]
3_C	Single_SC	0.20	[-0.38, 0.79]
3_D	Combi_ST	0.24	[-0.12, 0.61]
3_D	Single_ST	0.51	[-0.05, 1.07]
4_A	Doesnt_have_CBT	0.35	[0.10, 0.61]
4_A	Has_CBT	0.36	[0.02, 0.70]
4_B	Doesnt_have_HA	0.34	[-0.08, 0.75]
4_B	Has_HA	0.50	[0.13, 0.87]
4_C	Doesnt_have_SC	0.45	[0.18, 0.72]
4_C	Has_SC	0.22	[-0.10, 0.54]
4_D	Doesnt_have_ST	0.38	[0.11, 0.66]
4_D	Has_ST	0.32	[0.02, 0.62]
5	Brain	0.29	[-0.06, 0.64]
5	Brain_and_Ear	0.30	[-0.05, 0.65]
5	Ear	0.49	[0.14, 0.84]

Note. Values depict least-squares mean changes with 95% CI in square brackets. Objective 1: Single vs. Combination (all treatments); Objective 2: All treatments against each other; Objective 3: Single vs. Combination (separately); Objective 4: Treatments with vs. treatments without; Objective 5: Ear-mediated vs. brain-mediated vs. ear- and brain-mediated treatments. CBT = Cognitive-Behavioural Therapy; HA = Hearing Aids; SC = Structured Counselling; ST = Sound Therapy.

25. *Table S20: Secondary Outcome (WHO-QoL-Bref 2: Psychological Health) at Final Visit*

**Objective**	**Contrast**	**Change from baseline**	**95% CI**
1	Combination	0.36	[0.07, 0.65]
1	Single	0.36	[0.08, 0.65]
2	CBT	0.33	[-0.25, 0.92]
2	CBT + HA	0.75	[-0.35, 1.86]
2	CBT + SC	0.28	[-0.35, 0.91]
2	CBT + ST	0.35	[-0.27, 0.97]
2	HA	0.35	[-0.18, 0.89]
2	HA + SC	0.15	[-0.81, 1.10]
2	HA + ST	0.41	[-0.37, 1.20]
2	SC	0.30	[-0.28, 0.88]
2	SC + ST	0.35	[-0.19, 0.89]
2	ST	0.47	[-0.09, 1.02]
3_A	Combi_CBT	0.38	[-0.05, 0.81]
3_A	Single_CBT	0.33	[-0.26, 0.93]
3_B	Combi_HA	0.43	[-0.08, 0.94]
3_B	Single_HA	0.35	[-0.16, 0.87]
3_C	Combi_SC	0.30	[-0.08, 0.68]
3_C	Single_SC	0.30	[-0.29, 0.89]
3_D	Combi_ST	0.36	[-0.01, 0.73]
3_D	Single_ST	0.47	[-0.10, 1.04]
4_A	Doesnt_have_CBT	0.36	[0.11, 0.61]
4_A	Has_CBT	0.36	[0.02, 0.70]
4_B	Doesnt_have_HA	0.34	[-0.09, 0.76]
4_B	Has_HA	0.39	[0.03, 0.75]
4_C	Doesnt_have_SC	0.40	[0.14, 0.66]
4_C	Has_SC	0.30	[-0.02, 0.61]
4_D	Doesnt_have_ST	0.34	[0.06, 0.61]
4_D	Has_ST	0.39	[0.09, 0.70]
5	Brain	0.31	[-0.05, 0.66]
5	Brain_and_Ear	0.37	[0.01, 0.73]
5	Ear	0.41	[0.06, 0.75]

Note. Values depict least-squares mean changes with 95% CI in square brackets. Objective 1: Single vs. Combination (all treatments); Objective 2: All treatments against each other; Objective 3: Single vs. Combination (separately); Objective 4: Treatments with vs. treatments without; Objective 5: Ear-mediated vs. brain-mediated vs. ear- and brain-mediated treatments. CBT = Cognitive-Behavioural Therapy; HA = Hearing Aids; SC = Structured Counselling; ST = Sound Therapy.

26. Table S21: Secondary Outcome (WHO-QoL-Bref 3: Social Factors) at Final Visit

Objective	Contrast	Change from baseline	95% CI
1	Combination	0.17	[-0.26, 0.60]
1	Single	0.20	[-0.22, 0.63]
2	CBT	0.26	[-0.62, 1.14]
2	CBT + HA	0.49	[-1.14, 2.12]
2	CBT + SC	0.62	[-0.35, 1.59]
2	CBT + ST	0.14	[-0.76, 1.04]
2	HA	0.63	[-0.18, 1.43]
2	HA + SC	0.93	[-0.48, 2.34]
2	HA + ST	-0.09	[-1.27, 1.10]
2	SC	0.01	[-0.86, 0.88]
2	SC + ST	-0.38	[-1.19, 0.42]
2	ST	-0.09	[-0.93, 0.75]
3_A	Combi_CBT	0.39	[-0.23, 1.01]
3_A	Single_CBT	0.26	[-0.62, 1.14]
3_B	Combi_HA	0.38	[-0.40, 1.15]
3_B	Single_HA	0.63	[-0.16, 1.41]
3_C	Combi_SC	0.19	[-0.35, 0.73]
3_C	Single_SC	0.01	[-0.82, 0.84]
3_D	Combi_ST	-0.13	[-0.69, 0.43]
3_D	Single_ST	-0.09	[-0.97, 0.79]
4_A	Doesnt_have_CBT	0.08	[-0.31, 0.47]
4_A	Has_CBT	0.35	[-0.16, 0.86]
4_B	Doesnt_have_HA	0.30	[-0.34, 0.94]
4_B	Has_HA	0.50	[-0.07, 1.06]
4_C	Doesnt_have_SC	0.22	[-0.18, 0.62]
4_C	Has_SC	0.14	[-0.35, 0.62]
4_D	Doesnt_have_ST	0.43	[0.01, 0.84]
4_D	Has_ST	-0.12	[-0.58, 0.34]
5	Brain	0.29	[-0.24, 0.81]
5	Brain_and_Ear	0.06	[-0.47, 0.59]
5	Ear	0.20	[-0.32, 0.73]

Note. Values depict least-squares mean changes with 95% CI in square brackets. Objective 1: Single vs. Combination (all treatments); Objective 2: All treatments against each other; Objective 3: Single vs. Combination (separately); Objective 4: Treatments with vs. treatments without; Objective 5: Ear-mediated vs. brain-mediated vs. ear- and brain-mediated treatments. CBT = Cognitive-Behavioural Therapy; HA = Hearing Aids; SC = Structured Counselling; ST = Sound Therapy.

27. *Table S22: Secondary Outcome (WHO-QoL-Bref 4: Environment) at Final Visit*

Objective	Contrast	Change from baseline	95% CI
1	Combination	0.28	[-0.04, 0.61]
1	Single	0.35	[0.02, 0.67]
2	CBT	0.36	[-0.31, 1.02]
2	CBT + HA	0.57	[-0.72, 1.85]
2	CBT + SC	0.06	[-0.64, 0.75]
2	CBT + ST	0.46	[-0.21, 1.13]
2	HA	0.74	[0.14, 1.34]
2	HA + SC	0.66	[-0.44, 1.75]
2	HA + ST	0.35	[-0.53, 1.23]
2	SC	0.11	[-0.54, 0.76]
2	SC + ST	0.09	[-0.52, 0.71]
2	ST	0.17	[-0.47, 0.80]
3_A	Combi_CBT	0.31	[-0.13, 0.74]
3_A	Single_CBT	0.36	[-0.28, 0.99]
3_B	Combi_HA	0.50	[-0.11, 1.11]
3_B	Single_HA	0.74	[0.15, 1.33]
3_C	Combi_SC	0.16	[-0.27, 0.59]
3_C	Single_SC	0.11	[-0.56, 0.78]
3_D	Combi_ST	0.28	[-0.13, 0.69]
3_D	Single_ST	0.17	[-0.47, 0.81]
4_A	Doesnt_have_CBT	0.31	[0.02, 0.60]
4_A	Has_CBT	0.32	[-0.06, 0.71]
4_B	Doesnt_have_HA	0.29	[-0.20, 0.77]
4_B	Has_HA	0.62	[0.21, 1.02]
4_C	Doesnt_have_SC	0.43	[0.13, 0.73]
4_C	Has_SC	0.14	[-0.21, 0.50]
4_D	Doesnt_have_ST	0.37	[0.06, 0.67]
4_D	Has_ST	0.25	[-0.10, 0.59]
5	Brain	0.18	[-0.21, 0.57]
5	Brain_and_Ear	0.35	[-0.06, 0.75]
5	Ear	0.44	[0.04, 0.83]

Note. Values depict least-squares mean changes with 95% CI in square brackets. Objective 1: Single vs. Combination (all treatments); Objective 2: All treatments against each other; Objective 3: Single vs. Combination (separately); Objective 4: Treatments with vs. treatments without; Objective 5: Ear-mediated vs. brain-mediated vs. ear- and brain-mediated treatments. CBT = Cognitive-Behavioural Therapy; HA = Hearing Aids; SC = Structured Counselling; ST = Sound Therapy.

28. Table S23: Secondary Outcome (NRS 1: "How much of a problem is your tinnitus at present?") at Final Visit

Objective	Contrast	Change from baseline	95% CI
1	Combination	-0.53	[-0.71, -0.35]
1	Single	-0.49	[-0.67, -0.31]
2	CBT	-0.53	[-0.94, -0.11]
2	CBT + HA	-0.87	[-1.55, -0.20]
2	CBT + SC	-0.54	[-0.93, -0.14]
2	CBT + ST	-0.57	[-0.95, -0.19]
2	HA	-0.60	[-0.94, -0.27]
2	HA + SC	-0.49	[-1.09, 0.11]
2	HA + ST	-0.58	[-1.07, -0.08]
2	SC	-0.61	[-0.98, -0.25]
2	SC + ST	-0.39	[-0.72, -0.05]
2	ST	-0.24	[-0.60, 0.12]
3_A	Combi_CBT	-0.60	[-0.86, -0.33]
3_A	Single_CBT	-0.53	[-0.96, -0.09]
3_B	Combi_HA	-0.63	[-0.95, -0.31]
3_B	Single_HA	-0.60	[-0.91, -0.29]
3_C	Combi_SC	-0.46	[-0.69, -0.23]
3_C	Single_SC	-0.61	[-0.96, -0.27]
3_D	Combi_ST	-0.49	[-0.72, -0.26]
3_D	Single_ST	-0.24	[-0.61, 0.13]
4_A	Doesnt_have_CBT	-0.47	[-0.63, -0.31]
4_A	Has_CBT	-0.58	[-0.79, -0.36]
4_B	Doesnt_have_HA	-0.38	[-0.65, -0.12]
4_B	Has_HA	-0.62	[-0.85, -0.39]
4_C	Doesnt_have_SC	-0.52	[-0.69, -0.35]
4_C	Has_SC	-0.50	[-0.71, -0.30]
4_D	Doesnt_have_ST	-0.58	[-0.75, -0.41]
4_D	Has_ST	-0.42	[-0.61, -0.23]
5	Brain	-0.56	[-0.78, -0.34]
5	Brain_and_Ear	-0.52	[-0.74, -0.30]
5	Ear	-0.45	[-0.67, -0.23]

Note. Values depict least-squares mean changes with 95% CI in square brackets. Since the outcome is ordinally scaled, it was fitted as a sensitivity analysis with clmm of the ordinal package, as no differences were found to the mixed effect model, the results of the mixed effect model are reported here. Objective 1: Single vs. Combination (all treatments); Objective 2: All treatments against each other; Objective 3: Single vs. Combination (separately); Objective 4: Treatments with vs. treatments without; Objective 5: Ear-mediated vs. brain-mediated vs. ear-and brain-mediated treatments. CBT = Cognitive-Behavioural Therapy; HA = Hearing Aids; SC = Structured Counselling; ST = Sound Therapy.

29. *Table S24: Secondary Outcome (NRS 3: "How uncomfortable is your tinnitus at present?") at Final Visit*

Objective	Contrast	Change from baseline	95% CI
1	Combination	-1.68	[-2.13, -1.23]
1	Single	-1.25	[-1.70, -0.80]
2	CBT	-1.76	[-2.72, -0.80]
2	CBT + HA	-1.94	[-3.71, -0.17]
2	CBT + SC	-1.94	[-2.94, -0.93]
2	CBT + ST	-1.68	[-2.65, -0.72]
2	HA	-1.63	[-2.50, -0.77]
2	HA + SC	-1.22	[-2.76, 0.32]
2	HA + ST	-1.65	[-2.92, -0.38]
2	SC	-1.08	[-2.00, -0.16]
2	SC + ST	-1.55	[-2.42, -0.68]
2	ST	-0.55	[-1.44, 0.33]
3_A	Combi_CBT	-1.83	[-2.47, -1.18]
3_A	Single_CBT	-1.76	[-2.72, -0.80]
3_B	Combi_HA	-1.60	[-2.49, -0.71]
3_B	Single_HA	-1.63	[-2.52, -0.75]
3_C	Combi_SC	-1.65	[-2.25, -1.05]
3_C	Single_SC	-1.08	[-2.01, -0.15]
3_D	Combi_ST	-1.62	[-2.20, -1.03]
3_D	Single_ST	-0.55	[-1.45, 0.34]
4_A	Doesnt_have_CBT	-1.25	[-1.66, -0.85]
4_A	Has_CBT	-1.80	[-2.34, -1.27]
4_B	Doesnt_have_HA	-1.14	[-1.80, -0.47]
4_B	Has_HA	-1.62	[-2.22, -1.01]
4_C	Doesnt_have_SC	-1.46	[-1.87, -1.04]
4_C	Has_SC	-1.48	[-1.98, -0.98]
4_D	Doesnt_have_ST	-1.59	[-2.02, -1.16]
4_D	Has_ST	-1.31	[-1.80, -0.82]
5	Brain	-1.58	[-2.14, -1.02]
5	Brain_and_Ear	-1.60	[-2.16, -1.04]
5	Ear	-1.20	[-1.75, -0.64]

Note. Values depict least-squares mean changes with 95% CI in square brackets. Objective 1: Single vs. Combination (all treatments); Objective 2: All treatments against each other; Objective 3: Single vs. Combination (separately); Objective 4: Treatments with vs. treatments without; Objective 5: Ear-mediated vs. brain-mediated vs. ear- and brain-mediated treatments. CBT = Cognitive-Behavioural Therapy; HA = Hearing Aids; SC = Structured Counselling; ST = Sound Therapy.

30. *Table S25: Secondary outcome (NRS 4: "How annoying is your tinnitus at present?") at Final Visit*

Objective	Contrast	Change from baseline	95% CI
1	Combination	-1.53	[-1.99, -1.08]
1	Single	-1.18	[-1.62, -0.73]
2	CBT	-1.58	[-2.55, -0.62]
2	CBT + HA	-2.00	[-3.73, -0.27]
2	CBT + SC	-1.83	[-2.84, -0.81]
2	CBT + ST	-1.29	[-2.23, -0.36]
2	HA	-1.63	[-2.49, -0.78]
2	HA + SC	-1.68	[-3.21, -0.16]
2	HA + ST	-1.16	[-2.42, 0.09]
2	SC	-1.03	[-1.97, -0.10]
2	SC + ST	-1.48	[-2.34, -0.63]
2	ST	-0.47	[-1.35, 0.42]
3_A	Combi_CBT	-1.61	[-2.26, -0.97]
3_A	Single_CBT	-1.58	[-2.55, -0.62]
3_B	Combi_HA	-1.55	[-2.43, -0.66]
3_B	Single_HA	-1.63	[-2.53, -0.73]
3_C	Combi_SC	-1.64	[-2.24, -1.04]
3_C	Single_SC	-1.03	[-1.96, -0.10]
3_D	Combi_ST	-1.35	[-1.93, -0.78]
3_D	Single_ST	-0.47	[-1.36, 0.43]
4_A	Doesnt_have_CBT	-1.20	[-1.61, -0.78]
4_A	Has_CBT	-1.60	[-2.13, -1.08]
4_B	Doesnt_have_HA	-1.14	[-1.81, -0.46]
4_B	Has_HA	-1.59	[-2.19, -0.98]
4_C	Doesnt_have_SC	-1.28	[-1.69, -0.87]
4_C	Has_SC	-1.46	[-1.98, -0.94]
4_D	Doesnt_have_ST	-1.56	[-1.99, -1.13]
4_D	Has_ST	-1.09	[-1.58, -0.61]
5	Brain	-1.47	[-2.02, -0.92]
5	Brain_and_Ear	-1.50	[-2.05, -0.94]
5	Ear	-1.07	[-1.62, -0.52]

Note. Values depict least-squares mean changes with 95% CI in square brackets. Objective 1: Single vs. Combination (all treatments); Objective 2: All treatments against each other; Objective 3: Single vs. Combination (separately); Objective 4: Treatments with vs. treatments without; Objective 5: Ear-mediated vs. brain-mediated vs. ear- and brain-mediated treatments. CBT = Cognitive-Behavioural Therapy; HA = Hearing Aids; SC = Structured Counselling; ST = Sound Therapy.

31. *Table S26: Secondary outcome (NRS 5: "How easy is it for you to ignore your tinnitus at present?") at Final Visit*

Objective	Contrast	Change from baseline	95% CI
1	Combination	-1.71	[-2.26, -1.16]
1	Single	-1.20	[-1.74, -0.66]
2	CBT	-1.67	[-2.82, -0.52]
2	CBT + HA	-2.41	[-4.51, -0.30]
2	CBT + SC	-1.92	[-3.12, -0.73]
2	CBT + ST	-1.75	[-2.88, -0.63]
2	HA	-1.29	[-2.33, -0.25]
2	HA + SC	-0.87	[-2.73, 0.98]
2	HA + ST	-2.18	[-3.69, -0.66]
2	SC	-1.18	[-2.27, -0.10]
2	SC + ST	-1.36	[-2.39, -0.32]
2	ST	-0.68	[-1.72, 0.36]
3_A	Combi_CBT	-1.92	[-2.71, -1.12]
3_A	Single_CBT	-1.67	[-2.84, -0.50]
3_B	Combi_HA	-1.85	[-2.88, -0.81]
3_B	Single_HA	-1.29	[-2.33, -0.25]
3_C	Combi_SC	-1.50	[-2.22, -0.79]
3_C	Single_SC	-1.18	[-2.26, -0.11]
3_D	Combi_ST	-1.66	[-2.35, -0.97]
3_D	Single_ST	-0.68	[-1.75, 0.39]
4_A	Doesnt_have_CBT	-1.21	[-1.69, -0.74]
4_A	Has_CBT	-1.84	[-2.49, -1.19]
4_B	Doesnt_have_HA	-0.95	[-1.73, -0.16]
4_B	Has_HA	-1.58	[-2.29, -0.87]
4_C	Doesnt_have_SC	-1.49	[-1.99, -0.99]
4_C	Has_SC	-1.41	[-2.01, -0.80]
4_D	Doesnt_have_ST	-1.52	[-2.05, -0.99]
4_D	Has_ST	-1.37	[-1.94, -0.81]
5	Brain	-1.58	[-2.26, -0.90]
5	Brain_and_Ear	-1.55	[-2.22, -0.88]
5	Ear	-1.21	[-1.87, -0.55]

Note. Values depict least-squares mean changes with 95% CI in square brackets. Objective 1: Single vs. Combination (all treatments); Objective 2: All treatments against each other; Objective 3: Single vs. Combination (separately); Objective 4: Treatments with vs. treatments without; Objective 5: Ear-mediated vs. brain-mediated vs. ear- and brain-mediated treatments. CBT = Cognitive-Behavioural Therapy; HA = Hearing Aids; SC = Structured Counselling; ST = Sound Therapy.

32. *Table S27: Secondary outcome (NRS 6: “How unpleasant is your tinnitus at present?”) at Final Visit*

Objective	Contrast	Change from baseline	95% CI
1	Combination	-1.37	[-1.83, -0.91]
1	Single	-1.16	[-1.61, -0.71]
2	CBT	-1.31	[-2.31, -0.31]
2	CBT + HA	-1.93	[-3.64, -0.23]
2	CBT + SC	-1.93	[-2.90, -0.97]
2	CBT + ST	-1.11	[-2.06, -0.16]
2	HA	-1.40	[-2.24, -0.55]
2	HA + SC	-1.32	[-2.81, 0.16]
2	HA + ST	-1.38	[-2.61, -0.15]
2	SC	-1.14	[-2.05, -0.24]
2	SC + ST	-1.00	[-1.83, -0.16]
2	ST	-0.80	[-1.65, 0.05]
3_A	Combi_CBT	-1.57	[-2.22, -0.92]
3_A	Single_CBT	-1.31	[-2.31, -0.31]
3_B	Combi_HA	-1.51	[-2.39, -0.64]
3_B	Single_HA	-1.40	[-2.29, -0.50]
3_C	Combi_SC	-1.40	[-1.98, -0.82]
3_C	Single_SC	-1.14	[-2.04, -0.25]
3_D	Combi_ST	-1.11	[-1.70, -0.52]
3_D	Single_ST	-0.80	[-1.67, 0.07]
4_A	Doesnt_have_CBT	-1.13	[-1.52, -0.73]
4_A	Has_CBT	-1.49	[-2.03, -0.95]
4_B	Doesnt_have_HA	-1.01	[-1.67, -0.36]
4_B	Has_HA	-1.46	[-2.05, -0.86]
4_C	Doesnt_have_SC	-1.22	[-1.64, -0.81]
4_C	Has_SC	-1.33	[-1.82, -0.83]
4_D	Doesnt_have_ST	-1.46	[-1.89, -1.03]
4_D	Has_ST	-1.02	[-1.49, -0.55]
5	Brain	-1.45	[-2.01, -0.89]
5	Brain_and_Ear	-1.18	[-1.74, -0.62]
5	Ear	-1.15	[-1.69, -0.61]

Note. Values depict least-squares mean changes with 95% CI in square brackets. Objective 1: Single vs. Combination (all treatments); Objective 2: All treatments against each other; Objective 3: Single vs. Combination (separately); Objective 4: Treatments with vs. treatments without; Objective 5: Ear-mediated vs. brain-mediated vs. ear- and brain-mediated treatments. CBT = Cognitive-Behavioural Therapy; HA = Hearing Aids; SC = Structured Counselling; ST = Sound Therapy.

33. Figure S6: Secondary Outcome (CGI-I) at Final Visit – Single vs. Combination (Objective 1)

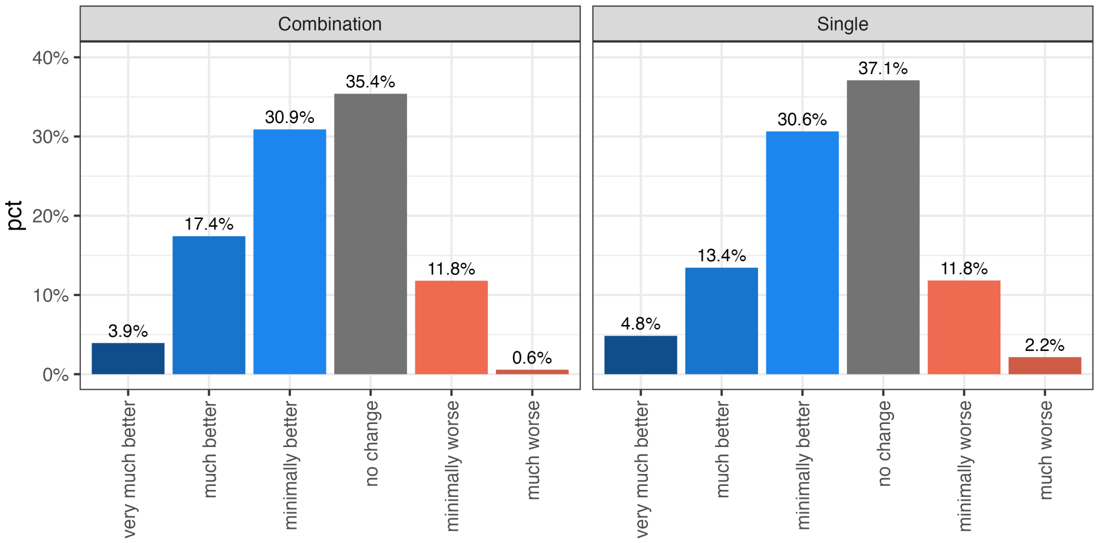

Note. Distribution (in percentage) of CGI-I (Clinical Global Impression Scale) at final visit for Objective 1.

34. *Figure S7: Secondary Outcome (CGI-I) at Final Visit – all Treatment Arms (Objective 2)*

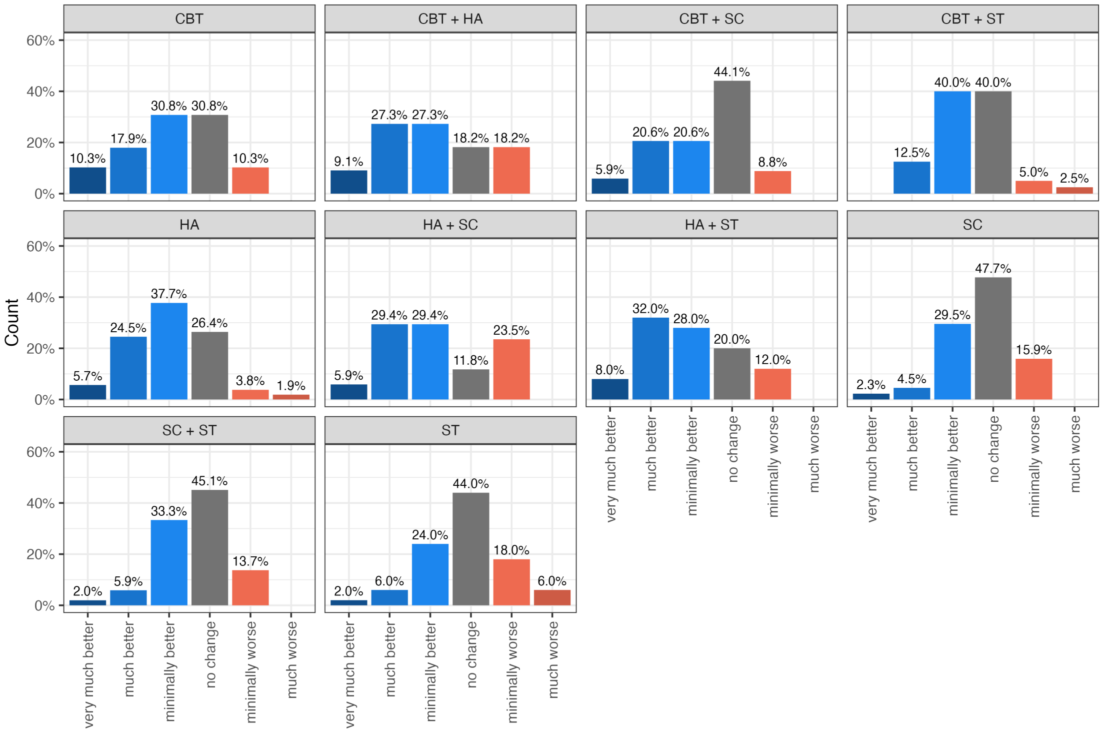

Note. Distribution (in percentage) of CGI-I (Clinical Global Impression Scale) at final visit for Objective 2. CBT = Cognitive-Behavioural Therapy; HA = Hearing Aids; SC = Structured Counselling; ST = Sound Therapy.

35. Figure S8: Secondary Outcome (CGI-I) at Final Visit – all Treatment Arms (Objective 2) in Patients With and Without Hearing Aid Indication

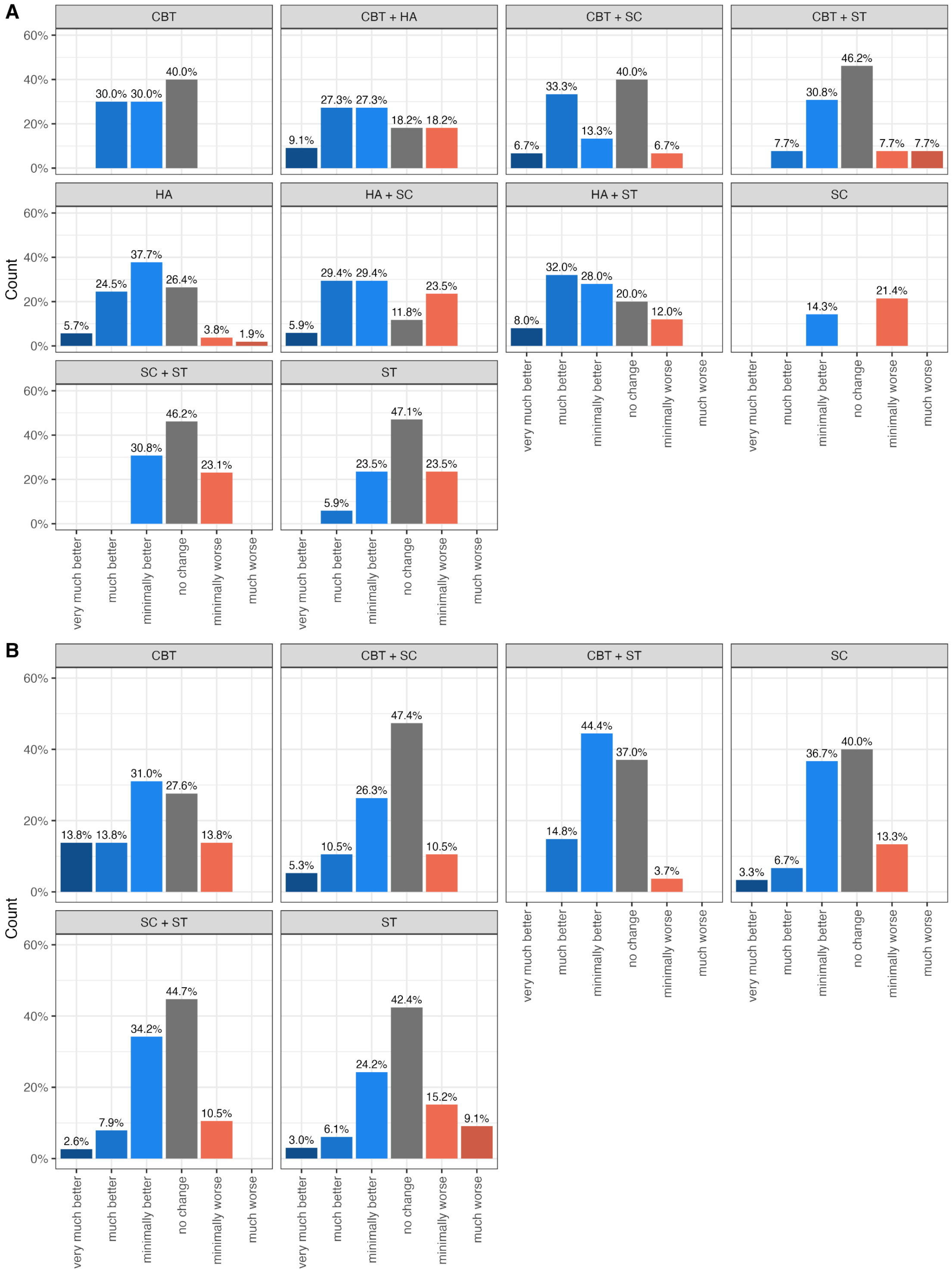

Note. Distribution (in percentage) of CGI-I (Clinical Global Impression Scale) at final visit for Objective 2 in patients (A) with hearing aid indication and (B) without hearing aid indication. CBT = Cognitive-Behavioural Therapy; HA = Hearing Aids; SC = Structured Counselling; ST = Sound Therapy.

36. *Figure S9: Secondary Outcome (CGI-I) at Final Visit – all Treatment Arms (Objective 2) in Patients with High and Low Tinnitus Distress Severity*

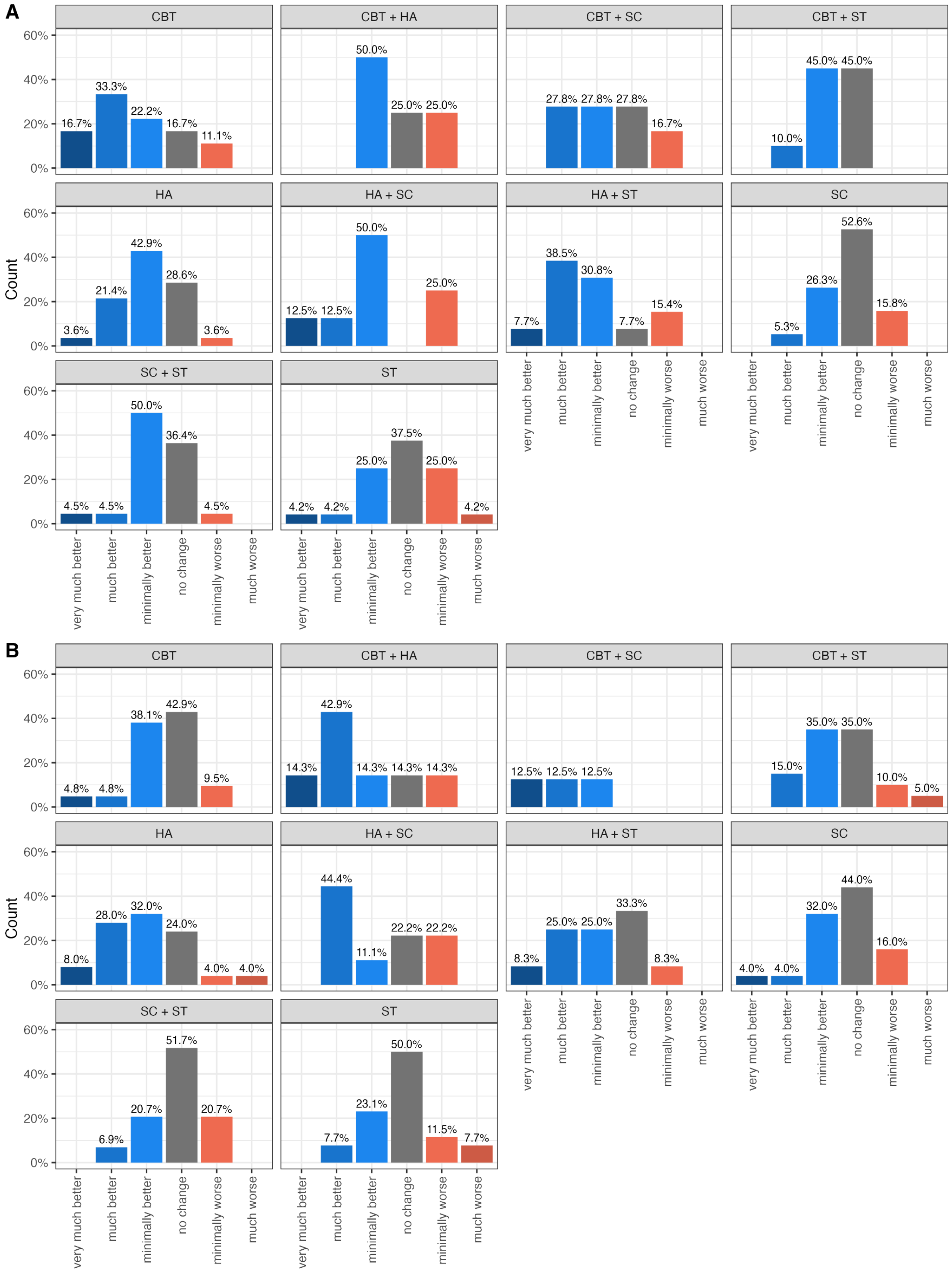

Note. Distribution (in percentage) of CGI-I (Clinical Global Impression Scale) at final visit for Objective 2 in patients (A) with High and (B) Low tinnitus distress severity. High tinnitus distress severity: THI $\geq$ 48 at Screening. Low tinnitus distress severity: THI < 48 at Screening. CBT = Cognitive-Behavioural Therapy; HA = Hearing Aids; SC = Structured Counselling; ST = Sound Therapy.

37. Table S28: Quantity and Type of Adverse Events per Treatment Arm

Treatment arm	***Adverse event***	***Treatment arm***	***Adverse event***
CBT	Reconstruction nasal septum	***CBT + HA***	Herpes zoster (thigh)
(n = 13)	Thoracic strain	***(n = 5)***	Skin Excision
	Additional burden due to husband's temporary absence		Mental breakdown
	Cold		Invasion Russia
	Diverticulitis disease		Ear pain due to hearing aids
	Torn tendon on the foot	***CBT + SC***	Vaccine reaction (Moderna)
	Sickness of spouse	***(n = 12)***	Lithotripsy
	Toothache		Total endoprosthesis hip
	Cold (suspected Covid-19 positive)		Leukoplakia vocal cord (surgery)
	Gastrointestinal infection		Chronic Fatigue Syndrome
	Worsening general health status		Vaccination reaction
	Death of close friend		Death of the cat, depressive symptoms of the significant other
	Worries about pet		Severe health problems of partner
HA	Mole removal		Angina
(n = 10)	Acute Urine Retention		Death of pet
	Tinnitus worsening		Heavy cold
	Retinal tear		Inheritance dispute with siblings
	Disease of the mother-in-law	***CBT + ST***	Common cold
	Loss of mother-in-law	***(n = 13)***	Covid-19 infection
	Urinary tract infection		Acute pain testis (left)
	Sleep problems		Covid-19 infection
	Covid-19 infection		Bereavement in the family
	Covid-19 infection		Cold
SC	Thyroid nodule (benign)		Mild temporary hearing loss
(n = 11)	Aggravation of tinnitus		Dizziness
	Diverticulitis		Worsening psychological health due to irritable colon or depression
	Tooth extraction		Sleep rhythm disturbed
	Septoplasty		Invasion Russia
	Stye		Covid-19 infection
	Professional reorientation		Tinnitus worsening
	Parents-in-law's dementia exacerbation	***HA + SC***	Broken foot
	Bicycle accident	***(n = 3)***	Ear infection due to hearing aid

	Additional task at work		Toothache
	Shoulder pain	***HA + ST*** <br>***(n = 5)***	Inflammation of the ear
ST <br>***(n = 15)***	Surgery hand (plate removal)		Frozen Shoulder
	Burglary		Travel problem on Airport
	Bicycle accident		Severe cold
	Influenza		Work stress
	Vaccine reaction (Covid-19)	***SC + ST*** <br>***(n = 11)***	Lymph node removal (groin)
	Depression		Forthcoming divorce
	Tooth infection and extraction		A lot of stress at the beginning of the school year
	Great workload and stress		Covid-19 infection
	Sprained foot		Migraine
	Disastrous vacation		Tinnitus worsening
	Cold		Tinnitus worsening
	Cold		Accident
	Persistent tiredness		Stress at work
	Gastrointestinal infection		Bicycle accident
	Tinnitus unchanged but more present and therefore more bothersome		Conflict with spouse

Note. Adverse events potentially associated with treatment were worsening of the tinnitus percept (6); worsening of psychological health (3); sleep problems (2); pain in the ear when wearing the hearing aid (1), ear infection (1), inflammation of the ear (1), dizziness (1), and mild transient hearing loss (1). CBT = Cognitive-Behavioural Therapy; HA = Hearing Aids; SC = Structured Counselling; ST = Sound Therapy.

38. *Table S29: Compliance to Treatments*

	**CBT**	**HA – left**	**HA – right**	**SC**	**ST**
Mean	7.5	5.0	5.3	47.1	108.6
Sd	3.7	3.1	3.3	32.0	149.9

Note. CBT (Cognitive Behavioural Therapy): number of CBT sessions attended. HA (hearing aids) – left: average daily wearing time of hearing aids left (in hours). HA – right: average daily wearing time of hearing aids right (in hours). SC (Structured Counseling): number of completed SC sections. ST (Sound Therapy): number of sounds played in ST.

39. *Table S30: Primary Outcome (THI) Objective 2: Post-hoc Comparisons (Baseline to Final Visit)*

Contrast (baseline – final visit)	Estimate	95% CI	p	p-adjusted
CBT - (CBT + HA)	1.62	[-7.47, 10.71]	.727	1.00
CBT - (CBT + SC)	-0.53	[-7.01, 5.96]	.874	1.00
CBT - (CBT + ST)	2.77	[-3.56, 9.11]	.391	1.00
CBT - HA	2.46	[-3.50, 8.42]	.419	1.00
CBT - (HA + SC)	-3.18	[-11.50, 5.15]	.454	1.00
CBT - (HA + ST)	3.93	[-3.43, 11.29]	.295	1.00
CBT - SC	4.86	[-1.26, 10.98]	.119	1.00
CBT - (SC + ST)	4.21	[-1.70, 10.11]	.162	1.00
CBT - ST	13.03	[6.85, 19.21]	< .001***	< .001***
(CBT + HA) - (CBT + SC)	-2.14	[-11.60, 7.31]	.656	1.00
(CBT + HA) - (CBT + ST)	1.16	[-8.30, 10.61]	.811	1.00
(CBT + HA) - HA	0.84	[-8.24, 9.92]	.856	1.00
(CBT + HA) - (HA + SC)	-4.80	[-15.57, 5.98]	.383	1.00
(CBT + HA) - (HA + ST)	2.31	[-7.81, 12.43]	.654	1.00
(CBT + HA) - SC	3.24	[-5.97, 12.45]	.490	1.00
(CBT + HA) - (SC + ST)	2.59	[-6.56, 11.74]	.579	1.00
(CBT + HA) - ST	11.41	[2.21, 20.62]	.015*	.675
(CBT + SC) - (CBT + ST)	3.30	[-3.38, 9.98]	.333	1.00
(CBT + SC) - HA	2.98	[-3.30, 9.26]	.352	1.00
(CBT + SC) - (HA + SC)	-2.65	[-11.13, 5.83]	.540	1.00
(CBT + SC) - (HA + ST)	4.46	[-3.02, 11.93]	.242	1.00
(CBT + SC) - SC	5.39	[-1.05, 11.83]	.101	1.00
(CBT + SC) - (SC + ST)	4.73	[-1.43, 10.90]	.132	1.00
(CBT + SC) - ST	13.56	[7.21, 19.90]	< .001***	< .001***
(CBT + ST) - HA	-0.32	[-6.22, 5.59]	.916	1.00
(CBT + ST) - (HA + SC)	-5.95	[-14.30, 2.40]	.162	1.00
(CBT + ST) - (HA + ST)	1.16	[-6.07, 8.38]	.753	1.00
(CBT + ST) - SC	2.09	[-3.94, 8.11]	.497	1.00
(CBT + ST) - (SC + ST)	1.43	[-4.47, 7.34]	.634	1.00
(CBT + ST) - ST	10.26	[4.32, 16.19]	.001**	.045*
HA - (HA + SC)	-5.63	[-13.75, 2.48]	.173	1.00
HA - (HA + ST)	1.48	[-5.56, 8.51]	.681	1.00

Contrast (baseline – final visit)	Estimate	95% CI	p	p-adjusted
HA - SC	2.40	[-3.36, 8.17]	.413	1.00
HA - (SC + ST)	1.75	[-3.84, 7.34]	.539	1.00
HA - ST	10.57	[4.78, 16.37]	< .001***	< .001***
(HA + SC) - (HA + ST)	7.11	[-2.05, 16.27]	.128	1.00
(HA + SC) - SC	8.04	[-0.09, 16.16]	.053	1.00
(HA + SC) - (SC + ST)	7.38	[-0.65, 15.42]	.072	1.00
(HA + SC) - ST	16.21	[8.06, 24.36]	< .001***	< .001***
(HA + ST) - SC	0.93	[-6.23, 8.09]	.799	1.00
(HA + ST) - (SC + ST)	0.28	[-6.75, 7.30]	.939	1.00
(HA + ST) - ST	9.10	[2.03, 16.16]	.012*	.540
SC - (SC + ST)	-0.65	[-6.39, 5.08]	.823	1.00
SC - ST	8.17	[2.29, 14.05]	.006**	.270
(SC + ST) - ST	8.82	[3.20, 14.45]	.002**	.090

Note. Objective 2: Pairwise comparison of treatments for the THI difference from baseline to final visit (post-hoc tests). The comparisons are based on a linear mixed effects model predicting the primary outcome by objective, time point (baseline, interim visit, final visit, and follow-up), and objective-by-time interaction as fixed effects, including centre and subject ID as random intercepts. The model was adjusted for the following covariates: age, sex, educational attainment, hearing aid indication, and PHQ-9 baseline scores. All comparisons are two-sided and p-values were adjusted for multiple comparisons using the Bonferroni method. CBT = Cognitive-Behavioural Therapy; HA = Hearing Aids; SC = Structured Counselling; ST = Sound Therapy. *p<0.05, **p<0.01, ***p<0.001.

40. Table S31: Sensitivity Analysis - Primary Outcome (THI) at Final Visit

Objective	Contrast	Change from baseline	95% CI
1	Combination	-15.10	[-17.96, -12.24]
1	Single	-11.53	[-14.34, -8.72]
2	CBT	-18.12	[-24.10, -12.14]
2	CBT + HA	-16.00	[-27.17, -4.82]
2	CBT + SC	-19.17	[-25.55, -12.79]
2	CBT + ST	-14.02	[-19.96, -8.08]
2	HA	-14.50	[-19.74, -9.27]
2	HA + SC	-20.67	[-29.92, -11.42]
2	HA + ST	-12.92	[-20.59, -5.25]
2	SC	-11.66	[-17.35, -5.97]
2	SC + ST	-12.48	[-17.77, -7.19]
2	ST	-3.03	[-8.39, 2.33]
3_A	Combi_CBT	-16.36	[-20.49, -12.23]
3_A	Single_CBT	-18.31	[-24.41, -12.21]
3_B	Combi_HA	-15.98	[-20.92, -11.04]
3_B	Single_HA	-14.54	[-19.50, -9.58]
3_C	Combi_SC	-16.02	[-19.74, -12.30]
3_C	Single_SC	-11.66	[-17.35, -5.96]
3_D	Combi_ST	-13.05	[-16.66, -9.44]
3_D	Single_ST	-3.01	[-8.52, 2.51]
4_A	Doesnt_have_CBT	-11.40	[-13.90, -9.00]
4_A	Has_CBT	-16.90	[-20.30, -13.50]
4_B	Doesnt_have_HA	-11.09	[-15.19, -7.00]
4_B	Has_HA	-15.27	[-18.92, -11.61]
4_C	Doesnt_have_SC	-12.31	[-14.91, -9.71]
4_C	Has_SC	-14.70	[-17.87, -11.54]
4_D	Doesnt_have_ST	-15.97	[-18.68, -13.27]
4_D	Has_ST	-10.06	[-13.02, -7.09]
5	Brain	-16.00	[-19.49, -12.50]
5	Brain_and_Ear	-14.43	[-17.92, -10.94]
5	Ear	-9.69	[-13.09, -6.29]

Note. Values depict least-squares mean changes with 95% CI in square brackets. Sensitivity analysis for the primary outcome was performed without imputation of the primary outcome. Objective 1: Single vs. Combination (all treatments); Objective 2: All treatments against each other; Objective 3: Single vs. Combination (separately); Objective 4: Treatments with vs. treatments without; Objective 5: Ear-mediated vs. brain-mediated vs. ear- and brain-mediated treatments. CBT = Cognitive-Behavioural Therapy; HA = Hearing Aids; SC = Structured Counselling; ST = Sound Therapy.

41. *Table S32: Sensitivity Analysis – Robustness Check of the Primary Outcome (THI) at Final Visit using different Imputation Methods*

Imputation method	Least-square mean change – single	Least-square mean change – combination	ß estimate	p value
Multilevel imputation	-11.7 [-14.4; -9.0]	-14.9 [-17.7; -12.1]	3.2 [0.2; 6.1]	0.034
No imputation	-11.5 [-14.3; -8.7]	-15.1 [-18.0; -12.2]	3.6 [0.5; 6.6]	0.022
Reference-based imputation (J2R)	-12.3 [-15.2; -9.4]	-14.5 [-17.1; -12.0]	2.3 [-1.7; 6.2]	0.252
Reference-based imputation (CIR)	-12.3 [-15.2; -9.4]	-14.5 [-17.2; -11.7]	2.2 [-2.0; 6.4]	0.292
Reference-based imputation (CR)	-12.3 [-15.2; -9.4]	-14.5 [-17.2; -11.8]	2.2 [-1.9; 6.3]	0.281
LOCF	-9.5 [-12.0; -7.1]	-12.6 [-15.1; -10.2]	3.1 [0.4; 5.7]	0.024

Note. Results of the primary objective (THI; single vs. combination treatments) under different assumptions of the missing data mechanism. Depicted are least-square mean changes with 95% CI in square brackets and the results (*ß* estimate with 95% CI in square brackets and *p* value) of the interaction effect (single vs. combination treatments at final visit vs. baseline). Reference-based imputation methods fill missing values following the distribution of a designated reference arm. We used the single treatment arm as reference (R package `RefBasedMI`). *Jump to reference* (J2R): As soon as a participant has a missing value, all future outcomes are set to match the reference arm's trajectory from that point onward. *Copy increments in reference* (CIR): Missing values are imputed by preserving the participant's last observed offset from the reference group but then applying the reference group's subsequent changes. *Copy reference* (CR): Missing values are imputed as if the participant were always in the reference group, effectively discarding any prior difference from the reference. *Last observation carried forward* (LOCF) replaces each participant's missing values with their most recent observed measurement, assuming that the outcome remains unchanged after the last observation (single imputation method).

42. Figure S10: Per Protocol (N = 185) Primary Outcome (THI)

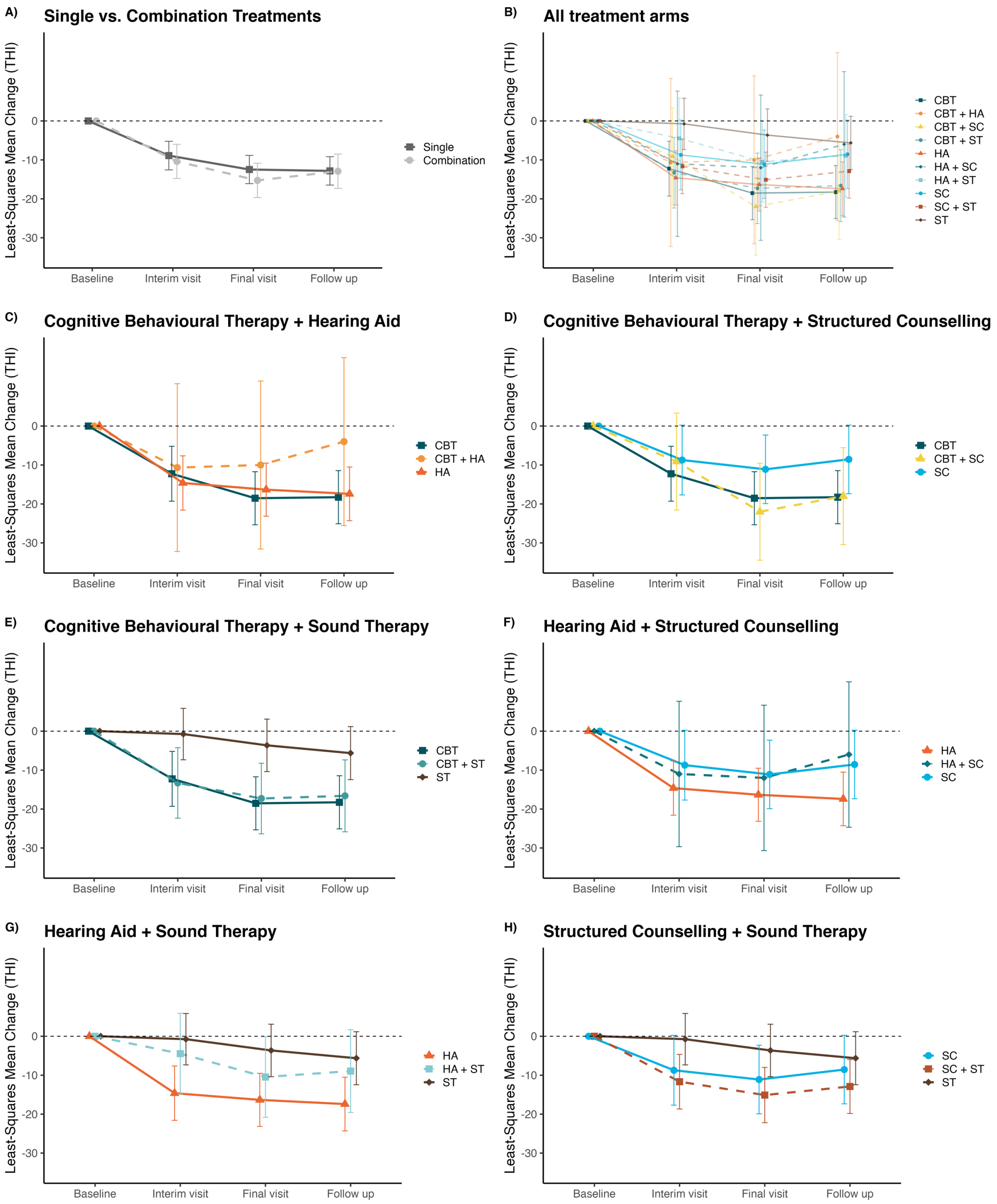

Note. Change from Baseline in THI total score to interim visit (6w), final visit (12w) and follow-up (36w) for A) single and combination treatments; B) all treatments; C) CBT + HA; D) CBT + SC; E) CBT + ST; F) HA + SC; G) HA + ST; and H) SC + ST. Total THI scores range from 0 to 100, with higher scores indicating greater severity of tinnitus. Error bars represent 95% confidence intervals.

43. Table S33: Per Protocol (N = 185) Primary Outcome (THI) at Final Visit

Objective	Contrast	Change from baseline	95% CI
1	Combination	-15.27	[-19.68, -10.86]
1	Single	-12.47	[-16.10, -8.84]
2	CBT	-18.53	[-25.35, -11.72]
2	CBT + HA	-10.00	[-31.55, 11.55]
2	CBT + SC	-22.00	[-34.44, -9.56]
2	CBT + ST	-17.29	[-26.35, -8.24]
2	HA	-16.33	[-23.15, -9.52]
2	HA + SC	-12.00	[-30.67, 6.67]
2	HA + ST	-10.46	[-20.82, -0.11]
2	SC	-11.11	[-19.91, -2.31]
2	SC + ST	-15.10	[-22.19, -8.01]
2	ST	-3.64	[-10.38, 3.10]
3_A	Combi_CBT	-18.00	[-25.29, -10.71]
3_A	Single_CBT	-18.53	[-25.70, -11.36]
3_B	Combi_HA	-10.70	[-18.27, -3.13]
3_B	Single_HA	-16.33	[-22.52, -10.15]
3_C	Combi_SC	-16.36	[-22.06, -10.66]
3_C	Single_SC	-11.11	[-19.68, -2.54]
3_D	Combi_ST	-14.70	[-19.71, -9.69]
3_D	Single_ST	-3.63	[-10.51, 3.24]
4_A	Doesnt_have_CBT	-11.38	[-14.75, -8.00]
4_A	Has_CBT	-18.27	[-23.16, -13.38]
4_B	Doesnt_have_HA	-10.84	[-16.53, -5.15]
4_B	Has_HA	-14.08	[-19.28, -8.88]
4_C	Doesnt_have_SC	-13.06	[-16.46, -9.66]
4_C	Has_SC	-14.76	[-19.69, -9.82]
4_D	Doesnt_have_ST	-16.19	[-20.08, -12.31]
4_D	Has_ST	-10.86	[-14.86, -6.86]
5	Brain	-16.74	[-21.73, -11.75]
5	Brain_and_Ear	-15.30	[-20.54, -10.06]
5	Ear	-9.98	[-14.37, -5.59]

Note. Values depict least-squares mean changes with 95% CI in square brackets. Objective 1: Single vs. Combination (all treatments); Objective 2: All treatments against each other; Objective 3: Single vs. Combination (separately); Objective 4: Treatments with vs. treatments without; Objective 5: Ear-mediated vs. brain-mediated vs. ear- and brain-mediated treatments. CBT = Cognitive-Behavioural Therapy; HA = Hearing Aids; SC = Structured Counselling; ST = Sound Therapy.

44. *Table S34: Per Protocol 2 (N = 155) Primary Outcome (THI) at Final Visit*

Objective	Contrast	Change from baseline	95% CI
1	Combination	-15.49	[-20.31, -10.68]
1	Single	-12.47	[-16.54, -8.40]
2	CBT	-19.05	[-27.26, -10.83]
2	CBT + HA	-10.00	[-31.74, 11.74]
2	CBT + SC	-22.75	[-36.06, -9.44]
2	CBT + ST	-18.29	[-28.35, -8.22]
2	HA	-17.48	[-24.73, -10.23]
2	HA + SC	-15.00	[-41.63, 11.63]
2	HA + ST	-9.64	[-20.99, 1.72]
2	SC	-10.38	[-19.79, -0.96]
2	SC + ST	-14.84	[-22.26, -7.41]
2	ST	-3.07	[-10.55, 4.40]
3_A	Combi_CBT	-18.72	[-26.52, -10.92]
3_A	Single_CBT	-19.05	[-27.56, -10.53]
3_B	Combi_HA	-10.38	[-19.03, -1.72]
3_B	Single_HA	-17.48	[-24.15, -10.82]
3_C	Combi_SC	-16.62	[-22.91, -10.34]
3_C	Single_SC	-10.38	[-19.76, -0.99]
3_D	Combi_ST	-14.66	[-20.15, -9.16]
3_D	Single_ST	-3.05	[-10.82, 4.72]
4_A	Doesnt_have_CBT	-11.52	[-15.20, -7.84]
4_A	Has_CBT	-18.87	[-24.49, -13.25]
4_B	Doesnt_have_HA	-11.03	[-17.36, -4.71]
4_B	Has_HA	-14.84	[-20.60, -9.07]
4_C	Doesnt_have_SC	-13.22	[-17.04, -9.41]
4_C	Has_SC	-14.73	[-20.07, -9.38]
4_D	Doesnt_have_ST	-16.62	[-20.97, -12.28]
4_D	Has_ST	-10.81	[-15.19, -6.44]
5	Brain	-16.62	[-22.33, -10.91]
5	Brain_and_Ear	-15.63	[-21.36, -9.90]
5	Ear	-10.32	[-15.14, -5.51]

Note. Values depict least-squares mean changes with 95% CI in square brackets. Per protocol 2: Additional exclusion of patients where time frame between baseline and final visit exceeded 18 weeks. Objective 1: Single vs. Combination (all treatments); Objective 2: All treatments against each other; Objective 3: Single vs. Combination (separately); Objective 4: Treatments with vs. treatments without; Objective 5: Ear-mediated vs. brain-mediated vs. ear- and brain-mediated treatments. CBT = Cognitive-Behavioural Therapy; HA = Hearing Aids; SC = Structured Counselling; ST = Sound Therapy.

45. Table S35: Clinical Sites and Principal Investigators

Country	Clinical Site	Principal Investigator
Belgium	Katholieke Universiteit Leuven, Leuven	Asst. Prof. Dr. Rilana Cima<br>Prof. Dr. Johan Vlaeyen
Germany	Tinnitus Center, Charité – Universitatsmedizin Berlin, corporate member of Freie Universität Berlin, Humboldt-Universität zu Berlin, and Berlin Institute of Health, Berlin	Prof. Dr. Birgit Mazurek<br>Dr. Benjamin Boecking
	University of Regensburg, Regensburg<br>(RCT coordinator)	Prof. Dr. Berthold Langguth<br>Prof. Dr. Martin Schecklmann<br>PD. Dr. Stefan Schoisswohl
Greece	Ethniko Kai Kapodistriako Panepistimo Athinon, Athens,	PD Dr. Dimitris Kikidis<br>Assoc. Prof. Dr. Athanasios Bibas
Spain	Hospital Universitario Virgen de las Nieves, Granada<br>Hospital Clinico Universitario San Cecilio, Granada	Prof. Dr. Jose Antonio López-Escámez<br>Assoc. Prof. Dr. Patricia Perez Carpena

46. *Table S36: Author Contributions*

Author name	Contribution	Author name	Contribution
Stefan Schoisswohl, Ph.D.	Conceptualisation; investigation; formal analysis; methodology; project administration; supervision; visualisation; writing – original draft	Juan Martin-Lagos, M.D.	Investigation; writing – review & editing
Laura Basso, Ph.D.	Data curation; formal analysis; methodology; visualisation; writing – original draft	Marta Martinez-Martinez, M.D., Ph.D.	Investigation; writing – review & editing
Jorge Simoes, Ph.D.	Data curation; formal analysis; methodology; writing – review & editing	Nicolas Muller-Locatelli, M.D.	Investigation; writing – review & editing
Milena Engelke, M.Sc.	Data curation; formal analysis; methodology; visualisation; writing – original draft	Patrick Neff, Ph.D.	Conceptualisation; Methodology; writing – review & editing
Berthold Langguth, M.D.	Conceptualisation; funding acquisition; methodology; supervision; writing – review & editing	Uli Niemann, Dr. Ing.	Data curation; formal analysis; methodology; writing – review & editing
Birgit Mazurek, M.D., Ph.D	Conceptualisation; funding acquisition; methodology; writing – review & editing	Patricia Perez-Carpena, M.D., Ph.D.	Investigation; writing – review & editing
Jose Antonio Lopez-Escamez, M.D., Ph.D.	Conceptualisation; funding acquisition; methodology; writing – review & editing	Rüdiger Pryss, Ph.D.	Funding acquisition; investigation; methodology; software; writing – review & editing
Dimitrios Kikidis, M.D., Ph.D.	Conceptualisation; funding acquisition; methodology; writing – review & editing	Clara Puga, M.Sc.	Data curation; formal analysis; methodology; writing – review & editing
Rilana Cima, Ph.D.	Conceptualisation; funding acquisition; methodology; writing – review & editing	Paula Robles-Bolivar, M.Sc.	Investigation; writing – review & editing
Alberto Bernal-Robledano, M.Sc.	Investigation; writing – review & editing	Matthias Rose, Ph.D.	Investigation; writing – review & editing
Benjamin Boecking, Ph.D., DClinPsy.	Conceptualisation; methodology; investigation; writing – review & editing	Martin Schecklmann, Ph.D.	Conceptualisation; investigation; methodology; writing – review & editing
Jan Bulla, Ph.D.	Formal analysis; writing – review & editing	Tabea Schiele, M.Sc.	Investigation; writing – review & editing
Christopher R. Cederroth, Ph.D.	Funding acquisition; resources; writing – review & editing	Miro Schleicher, M.Sc.	Data curation; formal analysis; methodology; writing – review & editing
Holger Crump, Dipl.-Soz.-Wiss	Conceptualisation; writing – review & editing	Johannes Schobel, Ph.D.	Investigation; software; writing – review & editing
Sam Denys, Ph.D.	Investigation; writing – review & editing	Myra Spiliopoulou, Dr. habil.	Funding acquisition; data curation, formal analysis; writing – review & editing
Alba Escalera-Balsera, M.Sc.	Investigation; writing – review & editing	Sabine Stark, Dipl.-Psych.	Investigation; writing – review & editing
Alvaro Gallego-Martinez, Ph.D.	Investigation; writing – review & editing	Susanne Staudinger, M.A.	Conceptualisation; project administration; investigation; writing – review & editing
Silvano Gallus, Ph.D.	Funding acquisition; writing – review & editing	Alexandra Stege, Ph.D.	Investigation; writing – review & editing
Hazel Goedhart, M.Sc	Conceptualisation; writing – review & editing	Beat Toedtli, Ph.D.	Formal analysis; validation; writing – review & editing
Leyre Hidalgo-Lopez, M.D.	Investigation; writing – review & editing	Ilias Trochidis, M.Sc.	writing – review & editing
Carlotta M. Jarach, M.Sc.	Data curation; formal analysis; methodology; writing – review & editing	Vishnu Unnikrishnan, M.Sc.	Data curation; formal analysis; methodology; writing – review & editing
Hafez Kader, M.Sc.	Data curation; formal analysis; methodology; writing – review & editing	Evgenia Vassou, M.Sc.	Investigation; writing – review & editing

Michael Koller, Ph.D.	Conceptualisation; writing – review & editing	Nicolas Verhaert, M.D, Ph.D.	Investigation; writing – review & editing
Alessandra Lugo, Ph.D.	Writing – review & editing	Carsten Vogel, M.Sc.	Investigation; methodology; software; writing – review & editing
Steven C. Marcrum, Ph.D.	Conceptualisation; investigation; writing – review & editing	Zoi Zachou, M.D.	Investigation; writing – review & editing
Nikos Markatos, B.Sc. (Honors)	Investigation; writing – review & editing	Winfried Schlee. Ph.D.	Conceptualisation; funding acquisition; formal analysis; methodology; project administration; supervision; visualisation; writing – original draft

Note. All authors fulfilled the necessary requirement for authorship and agreed to be fully accountable for all aspects of the present work. All authors read and approved the final manuscript.

47. Table S37: Criteria for HA indication

Frequency (Hz)	Minimum threshold (dB HL)	Maximum threshold (dB HL)
125	10	60
250	10	60
500	20	65
1000	25	70
2000	35	75
3000	40	80
4000	45	80
6000	45	80

Note. Minimum and maximum threshold (dB HL) per frequency used for the definition of HA indication. HA=Hearing aid.

48. *Table S38: Responsible Persons for the Conception of Treatments*

	**Profession**
**Cognitive Behavioural Therapy**	
Boecking, B.	Psychologist, Psychotherapist
Cima, R.	Psychologist, Psychotherapist
Schecklmann, M.	Psychologist, Psychotherapist
**Hearing Aid**	
Dettling-Papargyris, J.	Hearing aid acoustician
Kikidis, D.	Audiologist
Marcrum, S. C.	Audiologist
Mazurek, B.	ENT-Physician
Oppel, K.	Hearing aid acoustician
Schiele, T.	Doctoral student
Schlee, W.	Psychologist
**Structured Counselling**	
Engelke, M.	Psychologist
Schlee, W.	Psychologist
**Sound Therapy**	
Neff, P.	Psychologist
Schlee, W.	Psychologist

49. *Figure S11: Box-and-whisker Plot of Observed and Imputed THI Values*

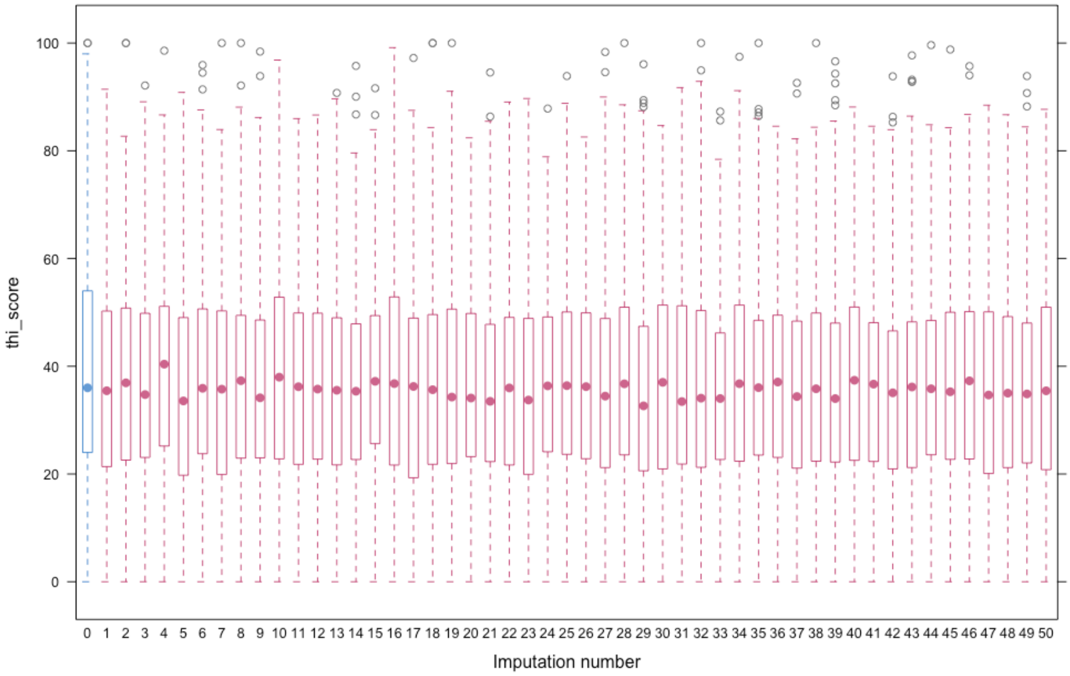

Note. Blue (0): Distribution of observed THI values (all visits). Red (1-50): Distribution of imputed THI values in all imputed data sets (all visits). THI = Tinnitus Handicap Inventory.

50. References

Probst T, Pryss RC, Langguth B, Spiliopoulou M, Landgrebe M, Vesala M, Harrison S, Schobel J, Reichert M, Stach M and Schlee W. Outpatient tinnitus clinic, self-help web platform, or mobile application to recruit tinnitus study samples? *Frontiers in Aging Neuroscience* 2017; *9*: 113.

51. Published Study Protocol & Statistical Analysis Plan

Study Protocol

Schoisswohl S, Langguth B, Schecklmann M, *et al.* Unification of Treatments and Interventions for Tinnitus Patients (UNITI): a study protocol for a multi-center randomized clinical trial. *Trials* 2021; 22: 875.

https://trialsjournal.biomedcentral.com/articles/10.1186/s13063-021-05835-z

Statistical Analysis Plan

Simoes JP, Schoisswohl S, Schlee W, *et al.* The statistical analysis plan for the unification of treatments and interventions for tinnitus patients randomized clinical trial (UNITI-RCT). *Trials* 2023; 24: 472

https://trialsjournal.biomedcentral.com/articles/10.1186/s13063-023-07303-2

52. Ethics approvals

Universität Regensburg

Ethikkommission
bei der Universität Regensburg

Ethikkommission · Universität Regensburg · 93040 Regensburg

Bezirksklinikum Regensburg, Klinik und
Poliklinik für Psychiatrie und Psychotherapie
Stefan Schoisswohl, BSc MSc
An der Steinernen Bank
93080 Pentling
Deutschland

Prof. Edward K. Geissler, PhD, Vorsitzender

Dr. iur. Frederike Seitz, M.A., Geschäftsführerin

Geschäftsstelle:
Telefon +49 941 943-5370
Telefax +49 941 943-5369
Postanschrift:
Universität Regensburg
ETHIKKOMMISSION
D-93040 Regensburg

ethikkommission@ur.de
http://ethikkommission.uni-regensburg.de

17.05.2021

Unser Zeichen: 20-1936_2-101

Beratung nach § 15 Abs. 1 Berufsordnung für die Ärzte Bayerns – Nachträgliche Änderung vom *10.05.2021*

für das

**Forschungsvorhaben**	**UNification of treatments and Interventions for TInnitus patients – Randomized Clinical Trial (UNITI-RCT)**
Antragssteller	Stefan Schoisswohl, BSc MSc

Die Ethikkommission nimmt die nachträglichen Änderungen am o.g. Forschungsvorhaben zur Kenntnis. Eine erneute inhaltliche Bewertung ist nach geltendem Recht nicht vorgesehen.

Diese Entscheidung erging durch den Vorsitzenden der Ethikkommission im Benehmen mit der Geschäftsstelle im beschleunigten Verfahren.

Es wird auf folgendes grundsätzlich hingewiesen:

Die ärztliche und juristische Verantwortung verbleibt beim Forscher und seinen Mitarbeitern.

Die Auflagen der Deklaration von Helsinki des Weltärztebundes in ihrer aktuellen Fassung hinsichtlich ethischen und rechtlichen Aspekten biomedizinischer Forschung am Menschen sind strikt zu beachten.

Die Ethikkommission erwartet bei Interventionsstudien, dass ihr alle schwerwiegenden oder unerwarteten unerwünschten Ereignisse (u.a. Todesfälle), die während der Studie auftreten und die Sicherheit der Studienteilnehmer oder die Durchführung der Studie beeinträchtigen können, unverzüglich schriftlich mitgeteilt werden. Dieses sollte in Verbindung mit einer Stellungnahme des Antragsstellers geschehen, ob aus seiner Sicht die Nutzen-Risiko-Relation des Vorhabens verändert ist.

Die Ethikkommission bittet darum, dass ihr der Abbruch oder Abschluss einer Studie mitgeteilt werden.

Dieses Schreiben ist mit den Studienunterlagen jederzeit sorgfältig aufzubewahren. Duplikate oder Abschriften dieses Schreibens können im Nachhinein nicht erstellt werden.

Auf die Rechtspflichten zum Umgang mit dienstlichem Schriftgut bzw. Urkunden wird verwiesen.

Die Ethikkommission bestätigt die Bearbeitung gemäß der GCP/ICH-Richtlinien.

Die Ethikkommission empfiehlt im Einklang mit der Deklaration von Helsinki nachdrücklich die Registrierung der Studie vor Studienbeginn in einem öffentlich zugänglichen Register, das die von der WHO geforderten Voraussetzungen erfüllt.

Falls kein gesetzlicher Kostenbefreiungstatbestand greift, wird ein gesonderter Kostenbescheid für die Gebühren und Auslagen der Ethikkommission ergehen.

Die Übermittlung personenbezogener Daten einschließlich DNA-tragender Biomaterialien in datenschutzrechtlich unsichere Drittstaaten, wie etwa die USA, bedarf einer gesonderten datenschutzrechtlichen Beurteilung und Risikoaufklärung.

Datenschutzrecht wird durch die Ethikkommission grundsätzlich nur kursorisch geprüft. Dieses Votum ersetzt mithin nicht die Konsultation des zuständigen Datenschutzbeauftragten.

Mit dem Urteil des Europäischen Gerichtshofs vom 16. Juli 2020 [Aktenzeichen C3-11/18] stellen die Regelungen des EU-US-Privacy Shield insbesondere vor dem Hintergrund des Clarifying Lawful Overseas Use of Data Act (CLOUD Act) bzw. des Foreign Surveillance Act (FISA) keinen tauglichen Rechtsrahmen mehr dar. Es sollte seitens der Verantwortlichen im Einzelfall geprüft werden, inwieweit personenbezogene/personenbeziehbare Daten (also auch i.S.d. Art. 4 Abs. 5 DSGVO pseudonymisierte Datensätze) rechtssicher entweder auf Basis geeigneter Garantien (etwa verbindlicher Unternehmensregeln, Standardvertragsklauseln oder auf Basis einer ausdrücklichen Einwilligung nach erfolgter Risiko-Aufklärung nach Art. 49 Abs. 1 lit. a) DSGVO) übermittelt werden können. Es bleiben v.a. hinsichtlich der Standardvertragsklauseln die Auswirkungen des Urteils und die voraussichtlich folgenden regulatorischen Leitlinien seitens der zuständigen Behörden aufmerksam zu verfolgen. Es ist daher den Sponsoren dringend zu raten, sich mit dem zuständigen Landesbeauftragten für den Datenschutz abzustimmen.

Mit freundlichen kollegialen Grüßen

Prof. Edward K. Geissler, PhD
Vorsitzender

Anlage

ΕΛΛΗΝΙΚΗ ΔΗΜΟΚΡΑΤΙΑ
1η Υ.ΠΕ ΑΤΤΙΚΗΣ
ΓΕΝ.ΝΟΣ/ΜΕΙΟ ΑΘΗΝΑΣ
«ΙΠΠΟΚΡΑΤΕΙΟ»

Ε.Σ. 47ο/26-1-2021

ΕΠΙΣΤΗΜΟΝΙΚΟ ΣΥΜΒΟΥΛΙΟ

ΠΡΑΚΤΙΚΟ 47ο/26-1-2021

Της Συνεδρίασης του Επιστημονικού Συμβουλίου του Γ.Ν.Α. «ΙΠΠΟΚΡΑΤΕΙΟ» στις 26/1/2021, ημέρα Τρίτη και ώρα έναρξης 13.30.

Με την υπ' αριθμ. 1034/22-1-2021 πρόσκληση του Προέδρου του Επιστημονικού Συμβουλίου κ. Δημητρίου Πετρά, κλήθηκαν και παρέστησαν στη Συνεδρίαση οι κ.κ.

1. Πετράς Δημήτριος
   Δ/ντής Νεφρολογικού Τμήματος
2. Πηρουνάκη Μαρία
   Δ/ντρια Παθολογίας
3. Μαρκογιαννάκης Χαρίδημος
   Επιμ. Β΄ Χειρουργικής
4. Γαλιατσάτος Νικόλαος
   Βιοχημικός πρ. Προϊστάμενος Βιοχημικού Τμ.
5. Κάπελλα Μαρία
   Δ/ντρια Νοσηλευτικής Υπηρεσίας
6. Παύλου Ευθυμία
   Τεχνολόγος ΤΕ Προϊσταμένη Ιατρικών Εργ/ρίων

Οι κ.κ. Βολτέας Σπυρίδων, Χρυσοβέργης Αριστείδης και Καραθανάσης Παναγιώτης απουσίασαν, λόγω κωλύματος.

Επίσης παρέστη η κα Ζέλκα Ευθυμία, ως Γραμματέας.

Μετά τη διαπίστωση της νόμιμης απαρτίας ο Πρόεδρος του Επιστημονικού Συμβουλίου, κηρύσσει την έναρξη της 47ης Συνεδρίασης με τα παρακάτω θέματα:

ΕΛΛΗΝΙΚΗ ΔΗΜΟΚΡΑΤΙΑ
1η Υ.ΠΕ ΑΤΤΙΚΗΣ
ΓΕΝ.ΝΟΣ/ΜΕΙΟ ΑΘΗΝΑΣ
«ΙΠΠΟΚΡΑΤΕΙΟ»

Ε.Σ. 47ο/26-1-2021

<u>ΕΠΙΣΤΗΜΟΝΙΚΟ ΣΥΜΒΟΥΛΙΟ</u>

Ε.Η.Δ. 12ο Έγκριση διεξαγωγής Κλινικής Έρευνας με κωδικό UNITI-RCTκαι τίτλο: ´´Ενοποίηση θεραπειών και παρεμβάσεων για ασθενείς με εμβοές (UNITI)-τυχαιοποιημένη κλινική δοκιμή (RCT)´´, με Επιστημονικά Υπεύθυνο τον Αν. Καθηγητή Ω.Ρ.Λ. κ. Αθανάσιο Μπίμπα.

Μετά από τη θετική εισήγηση της Επιτροπής Έρευνας κ´ Πρωτοκόλλων, υπό την Προεδρία του Καθηγητή κ. Ιωάννη Κοσκίνα

Ο μ ό φ ω ν α   ε γ κ ρ ί ν ε ι

Τη διεξαγωγή της Κλινικής Έρευνας με κωδικό UNITI-RCTκαι τίτλο: ´´Ενοποίηση θεραπειών και παρεμβάσεων για ασθενείς με εμβοές (UNITI)-τυχαιοποιημένη κλινική δοκιμή (RCT)´´, με Επιστημονικά Υπεύθυνο τον Αν. Καθηγητή Ω.Ρ.Λ. κ. Αθανάσιο Μπίμπα.

Ο ΠΡΟΕΔΡΟΣ
ΕΠΙΣΤΗΜΟΝΙΚΟΥ ΣΥΜΒΟΥΛΙΟΥ

ΔΗΜΗΤΡΙΟΣ ΠΕΤΡΑΣ

ΕΛΛΗΝΙΚΗ ΔΗΜΟΚΡΑΤΙΑ
1η Υ.ΠΕ ΑΤΤΙΚΗΣ
ΓΕΝ.ΝΟΣ/ΜΕΙΟ ΑΘΗΝΑΣ
«ΙΠΠΟΚΡΑΤΕΙΟ»

Ε.Σ. 47ο/26-1-2021

ΕΠΙΣΤΗΜΟΝΙΚΟ ΣΥΜΒΟΥΛΙΟ

Μη υπάρχοντος άλλου θέματος προς συζήτηση, ο κ. Πρόεδρος του Επιστημονικού Συμβουλίου λύει τη Συνεδρίαση.

Η ΓΡΑΜΜΑΤΕΑΣ Ε.Σ.

ΖΕΛΚΑ ΕΥΘΥΜΙΑ

Ο ΠΡΟΕΔΡΟΣ ΤΟΥ Ε.Σ.

ΔΗΜΗΤΡΙΟΣ ΠΕΤΡΑΣ

Προς Επιστημονικό Συμβούλιο

Γενικό Νοσοκομείο Αθηνών «Ιπποκράτειο»
Α Ωτορινολαρυγγολογική Κλινική Πανεπιστημίου Αθηνών

Κοινοποίηση: Διοίκηση Νοσοκομείου

Αθήνα, ~~18~~/12/ 2020

Θέμα: Αρχική Κατάθεση προς έγκριση για τη διεξαγωγή κλινικής έρευνας με κωδικό UNITI-RCT

Αριθμός πρωτοκόλλου: UNITI-RCT
Τίτλος πρωτοκόλλου: «Ενοποίηση θεραπειών και παρεμβάσεων για ασθενείς με εμβοές (UNITI) – τυχαιοποιημένη κλινική δοκιμή (RCT)»
Χορηγός: Δεν απαιτείται χορηγία για την παρούσα μελέτη. Το κάθε μεμονωμένο κέντρο είναι υπεύθυνο για τη διεξαγωγή της μελέτης σύμφωνα με τις κατευθυντήριες γραμμές για την ορθή κλινική πρακτική και τους τοπικούς κανονισμούς.

Αξιότιμοι Κύριοι,

Στα πλαίσια της διεξαγωγής της κλινικής έρευνας με κωδικό **UNITI-RCT** και τίτλο: **«Ενοποίηση θεραπειών και παρεμβάσεων για ασθενείς με εμβοές (UNITI) – τυχαιοποιημένη κλινική δοκιμή (RCT)»**, που πρόκειται να διεξαχθεί στην Α Ωτορινολαρυγγολογική Κλινική Πανεπιστημίου Αθηνών του νοσοκομείου με εμένα ως κύριο Ερευνητή, σας καταθέτουμε τα απαιτούμενα έγγραφα προς έγκριση .

Πρόκειται για μια πολυκεντρική τυχαιοποιημένη κλινικής δοκιμή (RCT) η οποία θα διεξαχθεί σύμφωνα με την Ορθή Κλινική Πρακτική (GCP) και την ειδική ανά χώρα νομοθεσία ως μέρος του προγράμματος UNITI σε πέντε διαφορετικά κλινικά κέντρα σε ολόκληρη την ΕΕ.

Ο κύριος στόχος της μελέτης αυτής είναι να διερευνηθεί κατά πόσο η συνδυαστική θεραπεία είναι πιο αποτελεσματική σε σύγκριση με τη μονοθεραπεία. Επιπρόσθετα, θα συγκριθούν οι εκβάσεις θεραπείας για κάθε επιμέρους παρέμβαση. Θα δημιουργηθούν αρκετές ομάδες παρέμβασης οι οποίες θα αναλυθούν βάσει του εάν έλαβαν μονοθεραπεία ή συνδυαστική θεραπεία, εάν έλαβαν μια συγκεκριμένη θεραπεία μεμονωμένα ή σε συνδυασμό με κάποια άλλη θεραπεία, εάν έλαβαν ή όχι μια συγκεκριμένη παρέμβαση (είτε σε συνδυασμό είτε μεμονωμένα) και εάν οι παρεμβάσεις που έλαβαν στόχευαν σε ένα ή δύο οργανικά επίπεδα (AS, ΚΝΣ). Μέσω ψυχολογικών, ακοολογικών, ηλεκτροφυσιολογικών και γενετικών δεδομένων θα ταυτοποιηθούν υπο-ομάδες προκειμένου ενδεχόμενα να προβλεφθεί η ανταπόκριση του ασθενούς σε ορισμένες παρεμβάσεις.Στη μελέτη θα ενταχθούν 500 άνδρες ή γυναίκες ασθενείς με εμβοές. Η μελέτη θα διεξαχθεί στη Γερμανία, την Ισπανία, ο Βέλγιο και την Ελλάδα

Η δοκιμή θα διαρκέσει περίπου 21 μήνες, συμπεριλαμβανομένης της στρατολόγησης, της παρέμβασης και της παρακολούθησης και ο προβλεπόμενος Αριθμός ασθενών για το κέντρο, είναι 100 ασθενείς.

Επισυνάπτονται τα κάτωθι:

1. UNITI-RCT – Σχέδιο κλινικής έρευνας
2. Έντυπο συναίνεσης μετά από ενημέρωση Έκδοση: 6.0 με ημερομηνία Ιούνιος 2020
3. Έντυπο συναίνεσης μετά από ενημέρωση Γενετική Ανάλυση Έκδοση: 4.0 με ημερομηνία Ιούνιος 2020

[illegible]ιημένη Διαδικασία λειτουργίας [illegible] 1.0 - Συλλογή αίματος και πλάσματος από ασθενείς με εμβοές

5. Τυποποιημένη Διαδικασία λειτουργίας έκδοση 1.0 - Εφαρμογή ηχοθεραπείας
6. Τυποποιημένη Διαδικασία λειτουργίας έκδοση 1.0 -Ηλεκτροφυσιολογικές μετρήσεις
7. Τυποποιημένη Διαδικασία λειτουργίας έκδοση 1.0 - Γνωστική συμπεριφορική θεραπεία για εμβοές (CBT4T)
8. Τυποποιημένη Διαδικασία λειτουργίας έκδοση 1.0 - Εφαρμογή δομημένης συμβουλευτικής
9. Τυποποιημένη Διαδικασία λειτουργίας έκδοση 1.0 - Ακοομετρία και Μέτρηση των Εμβοών
10. Τυποποιημένη Διαδικασία λειτουργίας έκδοση 2.0 - SOP εφαρμογής ακουστικού βαρηκοΐας*

Παρακαλώ πολύ στην γραπτή έγκριση να αναφέρονται τα ακόλουθα:

1. UNITI-RCT – Σχέδιο κλινικής έρευνας
2. Έντυπο συναίνεσης μετά από ενημέρωση Έκδοση: 6.0 με ημερομηνία Ιούνιος 2020
3. Έντυπο συναίνεσης μετά από ενημέρωση Γενετική Ανάλυση Έκδοση: 4.0 με ημερομηνία Ιούνιος 2020
4. Τυποποιημένη Διαδικασία λειτουργίας έκδοση 1.0 - Συλλογή αίματος και πλάσματος από ασθενείς με εμβοές
5. Τυποποιημένη Διαδικασία λειτουργίας έκδοση 1.0 - Εφαρμογή ηχοθεραπείας
6. Τυποποιημένη Διαδικασία λειτουργίας έκδοση 1.0 -Ηλεκτροφυσιολογικές μετρήσεις
7. Τυποποιημένη Διαδικασία λειτουργίας έκδοση 1.0 - Γνωστική συμπεριφορική θεραπεία για εμβοές (CBT4T)
8. Τυποποιημένη Διαδικασία λειτουργίας έκδοση 1.0 - Εφαρμογή δομημένης συμβουλευτικής
9. Τυποποιημένη Διαδικασία λειτουργίας έκδοση 1.0 - Ακοομετρία και Μέτρηση των Εμβοών
10. Τυποποιημένη Διαδικασία λειτουργίας έκδοση 2.0 - SOP εφαρμογής ακουστικού βαρηκοΐας*
11. Τα ονοματεπώνυμα και επαγγέλματα των μελών του Επιστημονικού Συμβουλίου, οι οποίοι ενέκριναν τη διεξαγωγή της παρούσας μελέτης.
12. Επιβεβαίωση ότι όλα τα κατατεθέντα στοιχεία έχουν ληφθεί υπόψη

Το νοσοκομείο δεν θα επιβαρυνθεί οικονομικά από την διεξαγωγή της μελέτης.

Στη διάθεση σας για οποιαδήποτε πρόσθετη πληροφορία ή διευκρίνιση
Με εκτίμηση,

Αν. Καθηγητής Αθανάσιος Μπίμπας
Α Ωτορινολαρυγγολογική Κλινική Πανεπιστημίου Αθηνών

4. Τυποποιημένη Διαδικασία λειτουργίας έκδοση 1.0 - Συλλογή αίματος και πλάσματος από ασθενείς με εμβοές
5. Τυποποιημένη Διαδικασία λειτουργίας έκδοση 1.0 - Εφαρμογή ηχοθεραπείας
6. Τυποποιημένη Διαδικασία λειτουργίας έκδοση 1.0 -Ηλεκτροφυσιολογικές μετρήσεις
7. Τυποποιημένη Διαδικασία λειτουργίας έκδοση 1.0 - Γνωστική συμπεριφορική θεραπεία για εμβοές (CBT4T)
8. Τυποποιημένη Διαδικασία λειτουργίας έκδοση 1.0 - Εφαρμογή δομημένης συμβουλευτικής
9. Τυποποιημένη Διαδικασία λειτουργίας έκδοση 1.0 - Ακοομετρία και Μέτρηση των Εμβοών
10. Τυποποιημένη Διαδικασία λειτουργίας έκδοση 2.0 - SOP εφαρμογής ακουστικού βαρηκοΐας*

Παρακαλώ πολύ στην γραπτή έγκριση να αναφέρονται τα ακόλουθα:

1. UNITI-RCT – Σχέδιο κλινικής έρευνας
2. Έντυπο συναίνεσης μετά από ενημέρωση Έκδοση: 6.0 με ημερομηνία Ιούνιος 2020
3. Έντυπο συναίνεσης μετά από ενημέρωση Γενετική Ανάλυση Έκδοση: 4.0 με ημερομηνία Ιούνιος 2020
4. Τυποποιημένη Διαδικασία λειτουργίας έκδοση 1.0 - Συλλογή αίματος και πλάσματος από ασθενείς με εμβοές
5. Τυποποιημένη Διαδικασία λειτουργίας έκδοση 1.0 - Εφαρμογή ηχοθεραπείας
6. Τυποποιημένη Διαδικασία λειτουργίας έκδοση 1.0 -Ηλεκτροφυσιολογικές μετρήσεις
7. Τυποποιημένη Διαδικασία λειτουργίας έκδοση 1.0 - Γνωστική συμπεριφορική θεραπεία για εμβοές (CBT4T)
8. Τυποποιημένη Διαδικασία λειτουργίας έκδοση 1.0 - Εφαρμογή δομημένης συμβουλευτικής
9. Τυποποιημένη Διαδικασία λειτουργίας έκδοση 1.0 - Ακοομετρία και Μέτρηση των Εμβοών
10. Τυποποιημένη Διαδικασία λειτουργίας έκδοση 2.0 - SOP εφαρμογής ακουστικού βαρηκοΐας*
11. Τα ονοματεπώνυμα και επαγγέλματα των μελών του Επιστημονικού Συμβουλίου, οι οποίοι ενέκριναν τη διεξαγωγή της παρούσας μελέτης.
12. Επιβεβαίωση ότι όλα τα κατατεθέντα στοιχεία έχουν ληφθεί υπόψη

Το νοσοκομείο δεν θα επιβαρυνθεί οικονομικά από την διεξαγωγή της μελέτης.

Στη διάθεση σας για οποιαδήποτε πρόσθετη πληροφορία ή διευκρίνιση
Με εκτίμηση,

Αν. Καθηγητής Αθανάσιος Μπίμπας
Α Ωτορινολαρυγγολογική Κλινική Πανεπιστημίου Αθηνών

JUNTA DE ANDALUCIA CONSEJERÍA DE SALUD Y FAMILIAS

DICTAMEN ÚNICO EN LA COMUNIDAD AUTÓNOMA DE ANDALUCÍA

D/Dª: ANTONIO SALMERON GARCIA como secretario/a del CEIM/CEI Provincial de Granada

CERTIFICA

Que este Comité ha evaluado la propuesta del promotor/investigador (No hay promotor/a asociado/a) para realizar el estudio de investigación titulado:

TÍTULO DEL ESTUDIO:	Unificación de tratamientos e intervenciones para pacientes con tinnitus - UNification of treatments and Interventions for TInnitus patients ,(UNITI)
Protocolo, Versión:	1
HIP, Versión:	1
CI, Versión:	1

Y que considera que:

Se cumplen los requisitos necesarios de idoneidad del protocolo en relación con los objetivos del estudio y se ajusta a los principios éticos aplicables a este tipo de estudios.

La capacidad del/de la investigador/a y los medios disponibles son apropiados para llevar a cabo el estudio.

Están justificados los riesgos y molestias previsibles para los participantes.

Que los aspectos económicos involucrados en el proyecto, no interfieren con respecto a los postulados éticos.

Y que este Comité considera, que dicho estudio puede ser realizado en los Centros de la Comunidad Autónoma de Andalucía que se relacionan, para lo cual corresponde a la Dirección del Centro correspondiente determinar si la capacidad y los medios disponibles son apropiados para llevar a cabo el estudio.

Lo que firmo en Granada a 28/01/2021

D/Dª. ANTONIO SALMERON GARCIA, como Secretario/a del CEIM/CEI Provincial de Granada

Código Seguro De Verificación:	919efafa5c41c421c6bd4888cff8f4a36303e263	Fecha	28/01/2021
Normativa	Este documento incorpora firma electrónica reconocida de acuerdo a la Ley 59/2003, de 19 de diciembre, de firma electrónica.		
Firmado Por	Antonio Salmeron Garcia		
Url De Verificación	https://www.juntadeandalucia.es/salud/portaldeetica/xhtml/ayuda/verificarFirmaDocumento.iface/code/919efafa5c41c421c6bd4888cff8f4a36303e263	Página	1/3

CERTIFICA

Que este Comité ha ponderado y evaluado en sesión celebrada el 26/01/2021 y recogida en acta 1/21 la propuesta del/de la Promotor/a (No hay promotor/a asociado/a), para realizar el estudio de investigación titulado:

TÍTULO DEL ESTUDIO:	Unificación de tratamientos e intervenciones para pacientes con tinnitus - UNification of treatments and Interventions for TInnitus patients ,(UNITI)
Protocolo, Versión:	1
HIP, Versión:	1
CI, Versión:	1

Que a dicha sesión asistieron los siguientes integrantes del Comité:

Presidente/a

D/Dª. AURORA BUENO CAVANILLAS

Vicepresidente/a

D/Dª. Paloma Muñoz de Rueda

Secretario/a

D/Dª. ANTONIO SALMERON GARCIA

Vocales

D/Dª. PATRICIA GALVEZ MARTIN
D/Dª. Juan Ramón Delgado Pérez
D/Dª. Berta Gorlat Sánchez
D/Dª. José Dario Sánchez López
D/Dª. Sonia Dominguez Almendros
D/Dª. Juan Mozas Moreno
D/Dª. SALVADOR ARIAS SANTIAGO
D/Dª. MARIA ESPERANZA DEL POZO GAVILAN
D/Dª. Francisco OValle Ravassa
D/Dª. Esther Espínola García
D/Dª. ANTONIO MORALES ROMERO
D/Dª. MARTA CUADROS CELORRIO
D/Dª. MARIA ANGELES GARCIA LIROLA
D/Dª. Encarnación Martínez García
D/Dª. FRANCISCO LUIS MANZANO MANZANO
D/Dª. MIGUEL LÓPEZ GUADALUPE
D/Dª. JUAN ROMERO COTELO
D/Dª. MANUEL MARTIN DIAZ
D/Dª. ANGEL COBOS VARGAS
D/Dª. LUIS MIGUEL DOMENECH GIL
D/Dª. MARIA DEL ROCIO MORON ROMERO
D/Dª. Luis Javier Martínez González
D/Dª. JESÚS CARDONA CONTRERAS
D/Dª. Pilar Guijosa Campos
D/Dª. Miguel Álvarez López
D/Dª. RAFAEL MARIN JIMENEZ
D/Dª. JOAQUINA MARTINEZ GALAN
D/Dª. MARÍA DOLORES GARCÍA VALVERDE
D/Dª. ESTHER MOLINA RIVAS
D/Dª. ANTONIO JUAN PÉREZ FERNÁNDEZ
D/Dª. JUAN CARLOS NAVARRO BARRIOS

Código Seguro De Verificación:	919efafa5c41c421c6bd4888cff8f4a36303e263	Fecha	28/01/2021
Normativa	Este documento incorpora firma electrónica reconocida de acuerdo a la Ley 59/2003, de 19 de diciembre, de firma electrónica.		
Firmado Por	Antonio Salmeron Garcia		
Url De Verificación	https://www.juntadeandalucia.es/salud/portaldeetica/xhtml/ayuda/verificarFirmaDocumento.iface/code/919efafa5c41c421c6bd4888cff8f4a36303e263	Página	2/3

D/Dª. ANTONIO JIMENEZ PACHECO

Que dicho Comité, está constituido y actua de acuerdo con la normativa vigente y las directrices de la Conferencia Internacional de Buena Práctica Clínica.

Lo que firmo en Granada a 28/01/2021

Código Seguro De Verificación:	919efafa5c41c421c6bd4888cff8f4a36303e263	Fecha	28/01/2021
Normativa	Este documento incorpora firma electrónica reconocida de acuerdo a la Ley 59/2003, de 19 de diciembre, de firma electrónica.		
Firmado Por	Antonio Salmeron Garcia		
Url De Verificación	https://www.juntadeandalucia.es/salud/portaldeetica/xhtml/ayuda/verificarFirmaDocumento.iface/code/919efafa5c41c421c6bd4888cff8f4a36303e263	Página	3/3

Leuven, 15 Oct 2021

Ethics Committee
Research UZ/KU Leuven
Herestraat 49
B 3000 Leuven (Belgium)
Tel +32 16 34 86 00
Email : ec@uzleuven.be

dr. Rilana Cima

Our reference:	EudraCT-nr:	Belg. Regnr:
S65058		B3222021000553

UNification of treatments and Interventions for TInnitus patients – Randomized Clinical Trial

Positive advice in accordance with the law of 7 May 2004 on experiments on the human person

Dear colleague

The Ethics Committee Research (EC Research) of University Hospitals Leuven (UZ Leuven) has examined and discussed the above mentioned dossier at its meeting of 12 Jul 2021.

After having consulted the additional information and/or adapted documents relating to this dossier, EC Research considers that the proposed study, as described in the protocol, is scientifically relevant and ethically justified. It therefore gives on 15 Oct 2021 a favourable opinion of this study.

EC Research emphasizes the responsibility of the PI/promotor of this study concerning the privacy of the person/patient data in contacts with patients, or when viewing patient data, including the correct implementation thereof by coworkers and students. The PI/promotor is responsible for the implementation of the project proposal in accordance with applicable laws and regulations including, but not limited to, the EU regulation 2016/679 (General Data Protection Regulation), the Belgian Law on patients' rights of 22/8/2002, and the policy of the institution where the research will be carried out.

EC Research refers to the ICH/GCP guidelines on its website, and confirms that a GCP-training is required from each investigator. It is the responsibility of the principal investigator that each member of the study team has a valid GCP-certificate.

For the assessment of this dossier, documents/answers submitted on 22 Jun 2021, 24

Sep 2021, 08 Oct 2021 and 13 Oct 2021 have been taken into account.

The favourable advice concerns:

Protocol:
version 2.3 dd 13Oct2021
Informed Consent Form:
ICF v2.2 dd 01Oct2021 Nl + Fr + En
Other subject information documentation:
ATAQ version received dd 22Jun2021 Nl
BFI2-NL version received dd 22Jun2021 Nl
CGI version received dd 22Jun2021 Nl
ESIT-SQ version 1 dd Oct2018 Nl
FTQ version received dd 22Jun2021Nl
GÜF version received dd 22Jun2021 Nl
Mini-SOISES version received dd 22Jun2021 Nl
Mini-TQ version dd 02Feb2017
PHQ-9 version received dd 22Jun2021 Nl
TFI v4 post validation Nl
Tinnitus Handicap Inventaris version received dd 22Jun2021 Nl
Tinnitusernst version received dd 22Jun2021 Nl
TSCHQ : version received dd 22Jun2021 Nl
WHOQOL-BREF : version received dd 22Jun2021 Nl
Tinnitus daily diary version received dd 24Sep2021 Nl
Investigator's brochure/scientific leaflet:
SOP Electrophysiological Measurements v1.0
SOP Audiometry and Tinnitometry v1.0
SOP Blood and plasma collection from tinnitus patients v1.0
SOP Cognitive Behavioral Therapy for Tinnitus (CBT4T) v1.0
SOP Hearing Aid Fitting SOP* v2.0
SOP Sound Therapy App v1.0
SOP Structured Counselling App v1.0
Proof of "no-fault" insurance cover:
dd 01Jan2020 - 31Dec2022
Recruitment material:
recruitment flyer version 1.1 dd 23Sep2021 Nl
GDPR questionnaire:
dd 07Oct2021

EC Research confirms working in accordance with the ICH-GCP principles (International Conference on Harmonization Guidelines on Good Clinical Practice), the latest version of the Declaration of Helsinki, the Oviedo Convention on Human Rights and Biomedicine and applicable laws and regulations.

EC Research confirms that - in case of conflict of interest - involved members do not take part in the vote concerning the study.

List of members: see appendix.

<u>Points of concern:</u> (if applicable)

The conformity of translated documents compared to the Dutch documents, is the responsibility of the sponsor.

We would like to draw your attention to the fact that EC Research expects her initial comments to be taken into account ab initio at the next submission by the same sponsor.

Provided that there is a ***Clinical Trial Agreement****, the study can only start when the Clinical Trial Agreement has been approved and signed by the CEO of UZ Leuven (and/or by an authorized representative of KU Leuven R&D).*

Studies with investigational medicinal products and certain studies with "medical devices" should be submitted by the client (PI or sponsor) to the FAMHP (Federal Agency for Medicines and Health Products).

Studies with investigational medicinal products are only allowed to be conducted, provided that the minister (FAMHP) does not state objections within legal deadlines as described in art. 13 of the Belgian law of 7/5/2004 concerning experiments on human people.

Certain studies using medical devices are also covered by legal deadlines (KB of 17/3/2009). Please consult the FAMHP website for more information: www.fagg-afmps.be.

Research on embryos in vitro is covered by the law of May 11, 2003. Before the research project can start, such research also requires a positive advice of the Federal Committee for medical and scientific research on embryos in vitro.

Please take into account the regulations of the hospital concerning tissue management and the regulations of the law of December 19, 2008.
This favourable advice of EC Research does not imply that it will assume responsibility for the planned study. You will remain responsible for the study. In addition, you should ensure that your opinion as an involved researcher is reproduced in publications, reports for the government, etc. which are the result of this study. You are reminded that concerning clinical studies, any observed serious event needs to be reported immediately to the sponsor and the ethics committee, even if the causal relationship with the study is unclear.

The EC approval given for a specific project, is valid for one year. We request you to inform us if the study will not be initiated or if the study does not start within 1 year after approval.

If the study will not be terminated within a year, the ICH-GCP demands that an ***annual progress report*** *will be provided to EC Research.*

Finally, we request you to report the termination (early or planned) of the study within the legal deadlines and provide the ***Clinical Study Report*** *(CSR) to EC Research.*

In case of a clinical trial (EudraCT), please be informed that the results must be published in the European Clinical Trial Register. The report of these results can be sent to the EC Research as the CSR.

Yours sincerely,

Prof. Dr. Minne Casteels
Chair
Ethics Committee Research UZ Leuven

Cc:
FAMHP (Federal Agency for Medicines and Health Products)

CTC (Clinical Trial Center UZ Leuven)

List of members EC Research UZ/KU Leuven

Chair	prof. dr. Maria-Reinhilde Casteels	Clinical Pharmacology
Vice chair	prof. dr. Dominique Bullens	Paediatrics
	De heer Aernout De Raemaeker	Medical Legislation alternate
	De heer Jean-Jacques Derèze	Medical Legislation alternate
	De heer Mathijs Swaak	Healthy volunteer repres.
	Mevr. Angélique Rézer	Medical Legislation alternate
	Mevr. Annick Vanclooster	Nurse
	Mevr. Katelijne Van Overwalle	Pt representative (alternate)
	Mevr. Lia De Wilde	Pt representative (alternate)
	Mevr. Liliane Vandergeeten	Pt representative (alternate)
	Mevr. Marilien Vandeputte	Nurse
	Mevr. Michèle Dekervel	Medical Legislation alternate
	Mevr. Teresia De Fraye	Pt representative
	Mevr. Veerle Vanparys	Pharmacist (alternate)
	apr. Josse R. Thomas	Clinical Pharmacology
	dr. Kristel Van Landuyt	Reumatology
	dr. Lut De Groote	General Practitioner
	dr. Marleen Renard	Paediatrics
	prof. André Loeckx	Pt representative (alternate)
	prof. Ben Van Calster	Statistics
	prof. Guy Bosmans	Clinical Psycology (alternate)
	prof. Pascal Borry	Ethics
	prof. dr. Anne Smits	Paediatrics
	prof. dr. Anne Uyttebroeck	Paediatrics
	prof. dr. Ariel Alonso	Statistics (alternate)
	prof. dr. Benoit Nemery	Pneumology
	prof. dr. Gregor Verhoef	Haematology
	prof. dr. Jan Verhaegen	Laboratory Medicine
	prof. dr. Jan de Hoon	Clinical Pharmacology
	prof. dr. Karin Sipido	Experimental Cardiology
	prof. dr. Koen Luyckx	Clinical Psycology (alternate)
	prof. dr. Maria Schetz	Intensive care
	prof. dr. Simon Brumagne	Physiotherapy
	prof. dr. Xavier Bossuyt	Immunology
	prof. dr. apr. Erwin Dreesen	Pharmacist (alternate)
