# Supplementary material for: Single versus combination treatment in tinnitus: an international, multicentre, parallel-arm, superiority, randomised controlled trial
Source: Nat Commun. 2025 Nov 21;16:10510. doi: 10.1038/s41467-025-66165-1 (PMC12647723; doi:10.1038/s41467-025-66165-1)
Supplement: Supplementary file 2 — Reporting Summary [file 41467_2025_66165_MOESM2_ESM.pdf]

nature portfolio

Corresponding author(s): Stefan Schoisswohl

Last updated by author(s): 17.06.2025

Reporting Summary

Nature Portfolio wishes to improve the reproducibility of the work that we publish. This form provides structure for consistency and transparency in reporting. For further information on Nature Portfolio policies, see our Editorial Policies and the Editorial Policy Checklist.

Statistics

For all statistical analyses, confirm that the following items are present in the figure legend, table legend, main text, or Methods section.

n/a	Confirmed	
☐	☒	The exact sample size (*n*) for each experimental group/condition, given as a discrete number and unit of measurement
☐	☒	A statement on whether measurements were taken from distinct samples or whether the same sample was measured repeatedly
☐	☒	The statistical test(s) used AND whether they are one- or two-sided<br>*Only common tests should be described solely by name; describe more complex techniques in the Methods section.*
☐	☒	A description of all covariates tested
☐	☒	A description of any assumptions or corrections, such as tests of normality and adjustment for multiple comparisons
☐	☒	A full description of the statistical parameters including central tendency (e.g. means) or other basic estimates (e.g. regression coefficient) AND variation (e.g. standard deviation) or associated estimates of uncertainty (e.g. confidence intervals)
☐	☒	For null hypothesis testing, the test statistic (e.g. *F*, *t*, *r*) with confidence intervals, effect sizes, degrees of freedom and *P* value noted<br>*Give P values as exact values whenever suitable.*
☒	☐	For Bayesian analysis, information on the choice of priors and Markov chain Monte Carlo settings
☒	☐	For hierarchical and complex designs, identification of the appropriate level for tests and full reporting of outcomes
☐	☒	Estimates of effect sizes (e.g. Cohen's *d*, Pearson's *r*), indicating how they were calculated

Our web collection on statistics for biologists contains articles on many of the points above.

Software and code

Policy information about availability of computer code

Data collection	please see statistical analysis plan: https://trialsjournal.biomedcentral.com/articles/10.1186/s13063-023-07303-2
Data analysis	please see statistical analysis plan: https://trialsjournal.biomedcentral.com/articles/10.1186/s13063-023-07303-2

For manuscripts utilizing custom algorithms or software that are central to the research but not yet described in published literature, software must be made available to editors and reviewers. We strongly encourage code deposition in a community repository (e.g. GitHub). See the Nature Portfolio guidelines for submitting code & software for further information.

Data

Policy information about availability of data

All manuscripts must include a data availability statement. This statement should provide the following information, where applicable:

- Accession codes, unique identifiers, or web links for publicly available datasets
- A description of any restrictions on data availability
- For clinical datasets or third party data, please ensure that the statement adheres to our policy

De-identified data generated from this trial will initially be available only on request for researchers to reproduce results, later publicly via ZENODO. Current data availability will be reported on the UNITI website (https://uniti.tinnitusresearch.net/).

Human research participants

Policy information about studies involving human research participants and Sex and Gender in Research.

Reporting on sex and gender	No sex effect was found for the primary outcome (THI) when comparing single versus combination treatments for chronic tinnitus. 41.2 % of the participants were female, 58.8% were male.
Population characteristics	please see statistical analysis plan: https://trialsjournal.biomedcentral.com/articles/10.1186/s13063-023-07303-2
Recruitment	Potential candidates were recruited via media advertising (according to local regulations) as well as on an individual basis at the clinical sites through, e.g., information sheets, word of mouth, or in conversations with medical staff. In order to be eligible for participation, potential participants had to meet the following criteria as outlined in our study protocol: https://trialsjournal.biomedcentral.com/articles/10.1186/s13063-021-05835-z
Ethics oversight	The study was approved by local ethics committees of Granada, Athens, Leuven, Regensburg and Berlin (combined ethical approval for German sites).

Note that full information on the approval of the study protocol must also be provided in the manuscript.

Field-specific reporting

Please select the one below that is the best fit for your research. If you are not sure, read the appropriate sections before making your selection.

☒ Life sciences ☐ Behavioural & social sciences ☐ Ecological, evolutionary & environmental sciences

For a reference copy of the document with all sections, see nature.com/documents/nr-reporting-summary-flat.pdf

Life sciences study design

All studies must disclose on these points even when the disclosure is negative.

Sample size	The sample size was determined a priori on an estimated effect size of 0.26, an alpha level of 5% and a power of 80% (two-sided test). Based on that, the necessary sample size is 468. Considering potential dropouts, the aim was to recruit a total sample size of N = 500.16<br>The statistical analysis was performed in the intention-to-treat (ITT) population of N = 461, including all randomised participants, regardless of compliance with the study protocol. For the primary analysis (combination against single treatments), we estimated that with a two-tailed alpha level of less than 0.05, the sample size of N = 461 provides the trial with 90% power to detect an effect size of 0.30 (lower end of 95% CI for effect size of behavioural therapy interventions according to the latest Cochrane Review on tinnitus).
Data exclusions	Data was excluded for the PP analiysis in case of protocol violation.
Replication	No replication was undertaken; study was a RCT
Randomization	After successful on-site screening, eligible participants were stratified in four equally sized strata based on their THI total score (low [< 48] and high [≥ 48] tinnitus-related handicap) and hearing aid indication (yes and no, criteria for hearing aid indication: Table S37). Criterion for low and high tinnitus-related handicap was defined based on historical data obtained from 837 patients at the clinical site in Regensburg with a median THI score of 48. Hearing aid indication criteria were specified by a group of international experts in the fields of audiology and otolaryngology (see Table S38). Participants were then randomised to one of ten treatment arms comprised of single (CBT, HA, SC, ST) and combination interventions (CBT+HA, CBT+SC, CBT+ST, HA+SC, HA+ST, SC+ST) under consideration of the stratification group. Patients from the two strata without hearing aid indication were not randomised in treatment groups that comprised HA treatment. The stratification according to tinnitus-related handicap was performed to ensure an equal representation of patients with high and low tinnitus distress in different treatment arms and thus avoid potential misinterpretations of our findings due to large differences in baseline tinnitus severity across treatment arms. Randomisation was conducted at each clinical site with an interactive web response system developed together with biostatisticians from the contract research organization Excelya (www.excelya.com). Excelya was further responsible to monitor all randomisation proceedings.
Blinding	All statistical analysis were performed blinded to treatment allocation.

Reporting for specific materials, systems and methods

We require information from authors about some types of materials, experimental systems and methods used in many studies. Here, indicate whether each material, system or method listed is relevant to your study. If you are not sure if a list item applies to your research, read the appropriate section before selecting a response.

Materials & experimental systems

n/a	Involved in the study	
x		Antibodies
x		Eukaryotic cell lines
x		Palaeontology and archaeology
x		Animals and other organisms
	x	Clinical data
x		Dual use research of concern

Methods

n/a	Involved in the study	
x		ChIP-seq
x		Flow cytometry
x		MRI-based neuroimaging

Clinical data

Policy information about clinical studies

All manuscripts should comply with the ICMJE guidelines for publication of clinical research and a completed CONSORT checklist must be included with all submissions.

Clinical trial registration	ClinicalTrials.gov Identifier: NCT04663828.
Study protocol	Protocol: https://trialsjournal.biomedcentral.com/articles/10.1186/s13063-021-05835-z<br>SAP: https://trialsjournal.biomedcentral.com/articles/10.1186/s13063-023-07303-2
Data collection	please see statistical analysis plan: https://trialsjournal.biomedcentral.com/articles/10.1186/s13063-023-07303-2
Outcomes	please see statistical analysis plan: https://trialsjournal.biomedcentral.com/articles/10.1186/s13063-023-07303-2
